# Controlling aggregation of cholesterol-modified DNA nanostructures

**DOI:** 10.1093/nar/gkz914

**Published:** 2019-10-23

**Authors:** Alexander Ohmann, Kerstin Göpfrich, Himanshu Joshi, Rebecca F Thompson, Diana Sobota, Neil A Ranson, Aleksei Aksimentiev, Ulrich F Keyser

**Affiliations:** 1 Cavendish Laboratory, University of Cambridge, JJ Thomson Avenue, Cambridge CB3 0HE, UK; 2 Max Planck Institute for Medical Research, Department of Cellular Biophysics, Jahnstraße 29, 69120 Heidelberg, Germany; 3 Department of Physics, University of Illinois at Urbana-Champaign, 1110 West Green Street, Urbana, IL 61801, USA; 4 Faculty of Biological Sciences, University of Leeds, Leeds LS2 9JT, UK; 5 Department of Physics and Beckman Institute for Advanced Science and Technology, University of Illinois at Urbana-Champaign, 1110 West Green Street, Urbana, IL 61801, USA

## Abstract

DNA nanotechnology allows for the design of programmable DNA-built nanodevices which controllably interact with biological membranes and even mimic the function of natural membrane proteins. Hydrophobic modifications, covalently linked to the DNA, are essential for targeted interfacing of DNA nanostructures with lipid membranes. However, these hydrophobic tags typically induce undesired aggregation eliminating structural control, the primary advantage of DNA nanotechnology. Here, we study the aggregation of cholesterol-modified DNA nanostructures using a combined approach of non-denaturing polyacrylamide gel electrophoresis, dynamic light scattering, confocal microscopy and atomistic molecular dynamics simulations. We show that the aggregation of cholesterol-tagged ssDNA is sequence-dependent, while for assembled DNA constructs, the number and position of the cholesterol tags are the dominating factors. Molecular dynamics simulations of cholesterol-modified ssDNA reveal that the nucleotides wrap around the hydrophobic moiety, shielding it from the environment. Utilizing this behavior, we demonstrate experimentally that the aggregation of cholesterol-modified DNA nanostructures can be controlled by the length of ssDNA overhangs positioned adjacent to the cholesterol. Our easy-to-implement method for tuning cholesterol-mediated aggregation allows for increased control and a closer structure–function relationship of membrane-interfacing DNA constructs — a fundamental prerequisite for employing DNA nanodevices in research and biomedicine.

## INTRODUCTION

Interfacing DNA nanostructures with biological systems has gained significant interest in recent years, due to the ability to design arbitrary shapes with immense structural control combined with the relative ease of modifying DNA with functional groups in a sequence-programmable manner (1,2). Particularly, studying and mimicking biological processes at cell membranes or model lipid bilayers has become a popular and successful application of DNA nanotechnology (3,4).

Lipophilic DNA-conjugates have been used to functionalize polymersomes (5), microfluidic droplets (6) and, most prominently, lipid vesicles and planar lipid bilayers (7). Herein, lipid–DNA linkers have been employed for drug delivery (8), biosensing (9) and to assemble size-controlled liposomes (10) or stimuli-responsive liposome networks (11,12). Furthermore, DNA nanopores have been designed to self-insert into lipid membranes and induce ionic currents (13), thereby mimicking natural protein ion channels across multiple scales ranging from a single DNA duplex (14), via small non-scaffolded constructs (5,15–17), to large-conductance DNA origami transmembrane porins (18,19). A DNA-built enzyme demonstrated the ability to alter a membrane's lipid composition (20), while other DNA-based mimics induce and control membrane bending (21,22), act as membrane scaffolding (23), and resemble SNARE fusion proteins (24,25). All these successful examples rely on the hydrophobic modification of DNA.

The key to anchoring negatively charged DNA in lipid membranes are covalently linked hydrophobic modifications, specifically positioned on the DNA constructs. Although a variety of membrane anchors such as azobenzene (12), porphyrin (14–16), ethyl phosphorothioate (17) or tocopherol (18) have been employed, cholesterol is by far the most prominent (5–9,11–13,19–22,25–39). Its abundance in natural cell membranes as well as the commercial availability of cholesterol-tagged DNA with a variety of linkers, render it an ideal functional group to interface DNA nanostructures with biological and artificial lipid membranes.

However, a fundamental challenge is the strong tendency of the amphiphilic DNA–cholesterol conjugates to aggregate (18,26,31–36), a major problem well-known from the study of membrane proteins. Precisely controlled hydrophobic aggregation can be exploited as a switching mechanism (33) or to grow DNA crystals (34). However, a rationally designed structure-function relationship requires well-defined monomeric constructs. Krishnan *et al.* designed a large DNA-built channel decorated with 57 hydrophobic anchors which demonstrated increased membrane interaction but aggregated in solution (18). Johnson-Buck *et al.* reported significantly reduced hybridization kinetics of cholesterol-modified ssDNA and clustering of DNA-cholesterol nanostructures forcing them to use fewer hydrophobic groups to reduce aggregation (26). Already two cholesterol moieties have been shown to induce oligomerization in the assembly of DNA origami blocks (31). Cholesterol-mediated aggregation was also observed to cause reduced membrane binding efficiencies (35). Even after successful insertion, DNA nanostructures with three cholesterol moieties have been reported to form aggregates in a lipid bilayer (36). Nevertheless, higher numbers of cholesterol tags on a DNA construct are desirable and often necessary to facilitate spontaneous membrane insertion (18). Their collective free energy gain upon membrane incorporation reduces the energy barrier for DNA nanostructures to self-insert into a lipid bilayer (19,40), thereby facilitating more efficient membrane interaction. This intricate balance between optimal insertion properties, facilitated by an increased number of hydrophobic moieties, while preventing aggregation is typically only found by time-consuming trial and error.

Previous approaches to prevent cholesterol-induced aggregation of DNA nanostructures were limited to reducing the number of cholesterol tags (26), switching to an alternative membrane anchoring strategy using streptavidin as a linker between biotinylated DNA and lipids (18), or employing surfactants (32). However, fewer cholesterol modifications diminish membrane interaction (19), a streptavidin–biotin linkage is not applicable for studying biological cell membranes and their mimics, and surfactants are likely to impair the structure and integrity of lipid bilayers.

To overcome this longstanding challenge, we provide systematic insight into the factors that govern the aggregation of cholesterol-modified ssDNA, dsDNA and DNA nanostructures. With non-denaturing (native) polyacrylamide gel electrophoresis (PAGE) and all-atom molecular dynamics (MD) simulations, we initially analyze the aggregation of cholesterol-tagged ssDNA (chol-DNA). We then establish a mechanism to effectively reduce cholesterol-mediated interactions by introducing ssDNA overhangs adjacent to the hydrophobic group. Using transmission electron microscopy (TEM), PAGE, dynamic light scattering (DLS) and confocal microscopy, we characterize a model DNA nanostructure and its aggregation behavior depending on the cholesterol number, modification position, and linker type. Finally, we show that ssDNA overhangs adjacent to the cholesterol group can be applied as a universal mechanism to control aggregation of cholesterol-modified DNA nanostructures.

## MATERIALS AND METHODS

### DNA nanostructure assembly

DNA nanostructures were designed using the caDNAno software (41). In order to maximize specificity of the different strands, sequences were optimized using the web-based DNA analysis tool NUPACK (42). Unmodified DNA oligonucleotides were purchased from Integrated DNA Technologies, Inc. (IDT), purified by standard desalting or PAGE, and delivered at 100 μM in IDTE (10 mM Tris, pH 8.0, 0.1 mM EDTA). Required DNA strands for a specific construct were mixed at a final concentration of 1 μM in TE20 buffer (10 mM Tris, 1 mM EDTA, 20 mM MgCl_2_, pH 8.0). The mixture was heated to 85°C for 5 min, subsequently cooled to 25°C over 18 h and finally stored at 4°C, as described previously (20). If not stated otherwise, 3′-modified cholesterol strands were purchased from IDT with a triethylene glycol (TEG) linker, while 5′-modified strands were acquired from biomers.net with either a TEG or a C6 linker. Chol-DNA strands were HPLC purified, delivered dry and only then diluted in Milli-Q water (Merck Millipore) to a concentration of 100 μM and stored at 4°C. Prior to addition to the assembly mix, chol-DNA strands were vortexed briefly, spun down in a tabletop centrifuge and heated to 55°C for 10 min. Chol-DNA was incorporated into the DNA nanostructure by adding it to the assembly mix at 1 μM final concentration (1:1 stoichiometric ratio toward unmodified strands) while the respective unmodified oligonucleotide with the same sequence was omitted.

### Non-denaturing polyacrylamide gel electrophoresis

Chemicals were acquired from Sigma-Aldrich/Merck KGaA and gels were run in a Mini-PROTEAN Tetra Cell (Bio-Rad). Gels were hand cast at a final concentration of 10% polyacrylamide supplemented with 0.5× Tris–borate–EDTA (TBE), pH 8.0 and 11 mM MgCl_2_. Per 15 ml gel mixture, 150 μl 10% (w/v) ammonium persulfate solution (aliquots kept at −20°C and freshly thawed each time) and 10 μl *N*,*N*,*N*′,*N*′-tetramethylethylenediamine (TEMED) were added to start the polymerization which was typically left to set for at least 1 h before running the gel. 2 μl of assembled DNA nanostructures at 1 μM were mixed with 0.4 μl custom-made 6× loading dye (6×: 15% Ficoll 400, 0.9% Orange G diluted in TE20) and 2 μl of the mixture were loaded into the well, as described previously (20). For chol-DNA only, 1 μl of 100 μM chol-DNA solution (pre-heated as described in DNA nanostructure assembly) was mixed with 9 μl Milli-Q water and 2 μl of loading dye (this time prepared in Milli-Q water instead of TE20) and between 1 and 3 μl of this mixture were loaded. For duplexes of DNA and chol-DNA, strands were mixed at a 1:1 stoichiometric ratio in 50 mM NaCl and left at room temperature for 30 min before loading. GeneRuler Low Range (Thermo Fisher Scientific Inc.) was employed as the reference DNA ladder (L). Gels were run in 0.5× TBE supplemented with 11 mM MgCl_2_ at 100 V for 90 min on ice which are typical conditions for the analysis of DNA nanostructures (43,44). DNA was stained by immersing the gel slab for 10 min in 3× GelRed (Biotium). Subsequent imaging was performed on a GelDoc-It™ (UVP). Fiji software was used to invert the gray scale and subtract the background using the integrated rolling ball method at a radius of 300 pixel (>30% of the shortest image dimension). All operations were homogeneously applied to the whole image. Using the gel analyzer tool, intensity plots per lane were generated. The areas under the monomer, dimer and multimer intensity peaks were then determined and normalized to their sum per lane. Normalization compensated for a possible variation in sample loading volume and therefore allowed the comparison of folding yields between different lanes.

### All-atom MD simulations

All-atom MD simulations were performed using the molecular dynamics program NAMD2 (45). Initial atomistic models of ssDNA and dsDNA molecules were constructed using the NAB utility of AMBERTOOLs (46). A cholesterol group was covalently linked to the terminal 3′ carbon atom of the DNA backbone using a TEG linker, as described previously (20). CHARMM General Force Field (CGenFF) parameters (47) were used to describe the cholesterol-TEG’s bonded (covalent bonds within the cholesterol-TEG and to the DNA) and non-bonded interaction with surrounding atoms of DNA, water and ions. Mg^2+^-hexahydrates were added to compensate the DNA’s negative charge and to adjust its molarity to ≈11 mM, as used in experiments. The system was submersed in a box of water using the TIP3P model in the Solvate plugin of VMD and 100 mM of NaCl were added using the Autoionize plugin of VMD (48). The dimensions of fully assembled systems of ssDNA and dsDNA were 8 × 8 × 8 nm^3^ and 9 × 9 × 9 nm^3^ containing approximately 50 000 and 70 000 atoms, respectively. Assembled systems were subjected to energy minimization using the conjugate gradient method that removed steric clashes between the solute and solvent atoms. Subsequently, systems were equilibrated at constant number of atoms (N), pressure (*P* = 1 bar) and temperature (*T* = 291 K), i.e. an NPT ensemble. We used isotropic pressure coupling along all the three dimensions. All-atom MD simulations were performed using periodic boundary conditions and the particle mesh Ewald (PME) method to calculate the long-range electrostatic interactions in NAMD2 (45). The Nose-Hoover Langevin piston (49,50) and Langevin thermostat (51) were used to maintain the system's constant pressure and temperature. CHARMM36 force field parameters (52,53) along with NBFIX modifications for non-bonded interaction (54,55) were used to describe interatomic potential among water, ions and nucleic acids. An 8–10–12 Å cutoff scheme was used to calculate van der Waals and short-range electrostatic forces. All simulations were performed using 2 fs time steps for integrating the equation of motion. SETTLE (56) algorithm was applied to maintain rigid water molecules and RATTLE (57) algorithms were used to constrain all other covalent bonds involving hydrogen atoms. 500 ns equilibrium MD simulations were performed for each system. To improve sampling of the conformational space, two sets of simulations for each system were performed. Coordinates of the system were saved at an interval of 19.2 ps simulation. Analysis and post-processing of simulation trajectories were performed using VMD and CPPTRAJ (58).

### Attachment of DNA duplex and nanostructures to lipid membranes

Small unilamellar vesicles (SUVs) were formed according to a previously published protocol (59). In brief, 99.5 mol% 1-palmitoyl-2-oleoyl-sn-glycero-3-phosphocholine (POPC, Avanti Polar Lipids, USA) and 0.5 mol% Atto488-labeled 1,2-dioleoyl-*sn*-glycero-3-phosphoethanolamine (Atto488-DOPE, ATTO-TEC GmbH, Germany) were mixed in chloroform. The lipid mixture was dried under a gentle stream of nitrogen and subsequently placed in a desiccator for 2 h. Lipids were resuspended in 10 mM Tris, pH 8.0, 0.1 mM EDTA at a concentration of 2.2 mM, vortexed for 10 min and extruded through a 50 nm polycarbonate membrane (Avanti Polar Lipids, USA).

SUVs (final lipid concentration: 1.1 mM) were mixed with the respective DNA duplexes or nanostructures (final concentration: 0.5 μM) in TE20 buffer. The mixture was encapsulated into surfactant-stabilized water-in-oil droplets via the aqueous inlet of a microfluidic PDMS-based T-junction device. The oil phase contained HFE-7500 fluorinated oil (3M, Germany) with 1.4 wt% perfluoropolyetherpolyethylene glycol (PFPE-PEG) block-copolymer fluorosurfactants (Ran Biotechnologies, Inc., USA) and 10.5 mM PFPE-carboxylic acid (Krytox, MW: 7000–7500 g mol^−1^, DuPont, Germany). Under these conditions, SUVs fuse at the droplet periphery and form a spherical supported lipid bilayer (droplet-stabilized GUV or dsGUV). The attachment of DNA nanostructures can now be monitored with fluorescence microscopy. For details, see (59–61).

### Confocal fluorescence imaging

Confocal fluorescence imaging was performed to monitor the attachment of the DNA duplexes and nanostructures to the lipid membranes of dsGUVs. For this purpose, a Leica SP5 confocal laser scanning microscope (Leica Microsystems GmbH, Germany) was used, equipped with an argon and a white light laser as well as a 40× water immersion objective (HC PL APO 40×/1.10 w, CORR CS2, Leica Microsystems GmbH, Germany). The pinhole aperture was set to one Airy Unit and experiments were performed at room temperature. The recorded images were brightness- and contrast-adjusted and analyzed with ImageJ (NIH, USA).

### Transmission electron microscopy

3 μl of DNA nanostructure solution (1 μM in TE20) were deposited onto an electron microscopy grid (Agar Scientific) with a continuous carbon layer treated by glow discharge before use (30 s, 10 mA). Sample was blotted away, and the surface of the grid washed twice with water and once with 1% (w/v) uranyl acetate staining solution. 3 μl staining solution was then incubated on the surface of the grid for 30 s, excess was blotted away. Grids were imaged with a FEI G2-Spirit 12 BioTWIN transmission electron microscope with a Gatan US1000XP 2k × 2k CCD. Image analysis was performed using Gwyddion software. The background was flattened (polynomial) and an intensity mask was applied to identify DNA nanostructure monomers. Using the integrated grain distribution analysis, ellipses were fitted to each masked area and their major and minor axes were interpreted as the lengths and widths of the DNA nanostructures. This method allowed for the analysis of 13–53 DNA nanostructures per TEM image.

### Dynamic light scattering (DLS)

DLS measurements were performed on a Zetasizer Nano ZSP (Malvern Panalytical) with an excitation wavelength of 633 nm. DNA nanostructures were measured at 25°C and a concentration of 1 μM in TE20 buffer.

## RESULTS

### Sequence-dependence of cholesterol-modified ssDNA aggregation

**Figure 1.**
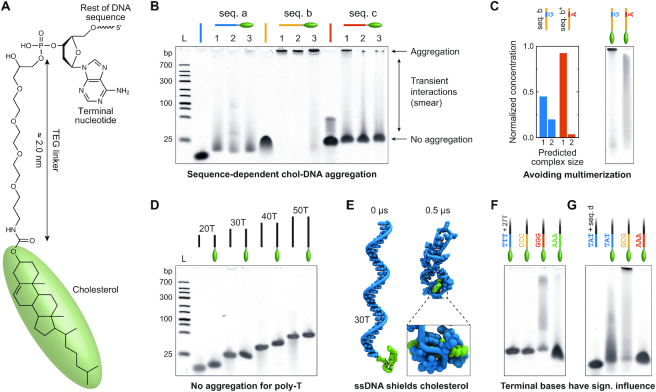
Sequence-dependent aggregation of cholesterol-modified ssDNA. (**A**) Chemical structure of cholesterol (green ellipsoid) covalently bound to the 3′ terminal nucleotide of ssDNA via a TEG linker. Its length was determined from the chemical structure in ChemBio3D Ultra (PerkinElmer). (**B**) Non-denaturing 10% PAGE of chol-DNA demonstrating different aggregation behaviors of three exemplary DNA sequences (seq. a: AAAACGCTAAGCCACCTTTAGATCCAAA, seq. b: AAACTCCCGGAGTCCGCTGCTGATCAAA, seq. c: GGATCTAAAGGACTTCTATCAAAGACGGGACGACTCCGGGAG; for further details see Supplementary Table S1). Numbers refer to different production batches and L to a DNA ladder. (**C**) Complex size (monomer / dimer) predicted by NUPACK shows reduced multimerization accompanied by reduced aggregation in PAGE if a specific base is changed from guanine to adenine in seq. b. (**D**) PAGE showing no aggregation of cholesterol-modified poly-thymine (poly-T) oligonucleotides of various lengths. (**E**) Instantaneous snapshots of an all-atom MD simulation of cholesterol-modified 30 thymidine ssDNA (30T) at the beginning (0 μs) and end (0.5 μs) of the simulation. The ssDNA wraps around the cholesterol group throughout the simulation. Water and ions are not shown for clarity. (**F**) Comparison of poly-T chol-DNA containing different terminal bases. Terminal guanines induce interactions between chol-DNA strands, visible as a smear in the gel. (**G**) Comparison of a mixed DNA sequence (seq. d: ACTGTCGAACATTTTTTGCCATAATA) showing significantly different aggregation behaviors if the three terminal bases are changed (+ TAT or GCG or AAA).

Most commonly, the cholesterol moiety is linked to the 3′ end of ssDNA via a TEG linker as shown in Figure 1A. To probe the aggregation behavior of chol-DNA, three different sequences (seq. a, b, c) that we employed in previous work (27), were analyzed with non-denaturing 10% PAGE (Figure 1B). Three separate production batches (1, 2, 3) were tested for each sequence to exclude a possible influence of the particular synthesis (see Supplementary Table S1 for sequences and batch details). The gel reveals a sequence-dependent aggregation behavior ranging from smeared bands, indicating transient interactions between DNA strands (seq. a), to complete aggregation in the well (seq. b), or a mixture of defined bands (indicating monomeric DNA strands) and aggregation (seq. c). Importantly, results are independent of the production batch (Figure 1B) illustrating that the behavior of cholesterol-modified ssDNA is sequence-dependent.

To determine the origin of the sequence-dependent behavior, we first tested if the particular, unmodified DNA sequences showed secondary structure formation using the web-based nucleic acid structure prediction tool NUPACK (42). While seq. a was predicted to remain exclusively monomeric, both seq. b and seq. c showed tendencies to form multimeric structures (Supplementary Figures S1 and S2). Other cholesterol-modified strands, even of low predicted dimerization tendency, similarly aggregated in the gel wells when modified with cholesterol (Supplementary Figure S3). We hypothesized that aggregation can be prevented by reducing multimer formation. Consequently, we altered a specific base which promoted aggregation in seq. b. A base change from guanine (seq. b) to adenine (seq. b*) increased the amount of predicted monomeric structures from 45% to 93% which completely averted aggregation in the well (Figure 1C and Supplementary Figure S2). However, a long smear still remains, indicating cholesterol-mediated interactions. Despite exclusively forming monomeric structures, seq. a also creates a smeared band. Therefore, multimerization can only partly explain the aggregation behavior of chol-DNA.

Since other secondary structures could possibly be the cause of aggregation, we tested poly-thymidine (poly-T) sequences as they are not prone to stable secondary formation (62). The gel in Figure 1D compares cholesterol-modified poly-T sequences ranging from 20 to 50 nucleotides, which are relevant lengths for the assembly of DNA nanostructures (43). As expected, longer DNA strands migrate slower in the gel. Furthermore, chol-DNA strands are slightly shifted to shorter run lengths compared to the corresponding unmodified strand, due to the increased hydrodynamic radius attributed to the cholesterol group. Notably, all cholesterol-modified strands form the same sharp bands as unmodified ones. This indicates that neither the interaction between the chol-DNA strands nor the interaction of the cholesterol group with the gel matrix has a significant effect on the migration of the DNA.

Next, we gained insight into the molecular interactions of the ssDNA with the cholesterol group by all-atom molecular dynamics (MD) simulations. We built an all-atom model of a 30 thymidine (30T) long ssDNA strand conjugated to cholesterol and submerged in electrolyte solution, and simulated the model at ambient temperature, pressure and ionic concentrations comparable to those used in experiments (see Materials and Methods). The snapshots in Figure 1E illustrate that throughout the 0.5 μs simulation, the cholesterol group is completely surrounded by the ssDNA, with the hydrophobic DNA bases facing the cholesterol moiety (Supplementary Movie M1). A second simulation, starting from the same initial configuration, yielded a similar result (Supplementary Figure S4). By wrapping around the cholesterol, the ssDNA effectively shields the cholesterol's hydrophobicity preventing cholesterol-mediated interactions, which agrees with the sharp bands observed in Figure 1D.

Lastly, we placed special emphasis on the influence of the three bases immediately adjacent to the cholesterol tag. PAGE results show no significant difference between three terminal thymine, adenine or cytosine nucleotides if the rest of the sequence is composed of poly-T (Figure 1F). However, terminal guanine nucleotides induced transient intermolecular interactions, visible as a completely smeared band (GGG). In contrast, all unmodified DNA strands showed no aggregation (Supplementary Figure S5). For chol-DNA of a mixed nucleotide sequence (seq. d), altering the terminal bases significantly affected the aggregation behavior (Figure 1G). The results ranged from a smeared band (TAT), to significant aggregation in the well (GCG), or no aggregation nor transient interactions at all (AAA). The behavior was independent of the supplier (IDT or biomers.net) or storage conditions before analysis, and all unmodified DNA strands show no aggregation themselves (Supplementary Figure S6). Furthermore, all sequences had almost no tendency to form dimers (≤0.7%), and secondary structures were similar with a low free energy (≈1 k_B_T; Supplementary Figure S7). These results show that particularly bases preceding the cholesterol modification present a significant influence on the sequence-dependent aggregation of chol-DNA. Analogous MD simulations as employed for the 30T sequence were performed on seq. d terminating with GCG or with AAA. While they demonstrate a similar shielding of the cholesterol moieties, the microsecond time scale of those simulations was too short to explain the experimentally observed difference in aggregation.

It is important to note that the sequence-dependent, cholesterol-mediated aggregation of chol-DNA was completely concealed in a control experiment with denaturing PAGE using urea (Supplementary Figure S8). This agrees with previous results on the masking of cholesterol-mediated aggregation if denaturing PAGE is employed (26). Thus, native (non-denaturing) PAGE is crucial for analyzing the influence of modifying DNA nanostructures with cholesterol and is therefore employed in this work.

### Controlling cholesterol-mediated DNA duplex aggregation by adjacent ssDNA

**Figure 2.**
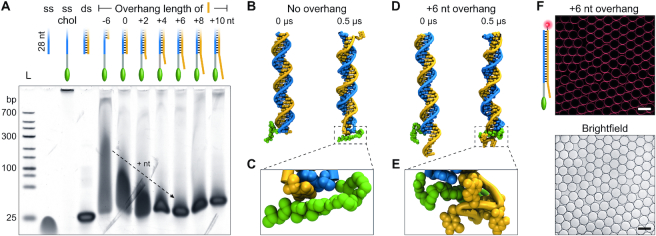
Preventing aggregation by adjacent ssDNA overhangs. (**A**) PAGE showing cholesterol-modified ssDNA (ss chol, blue) on its own and hybridized to a complementary strand (orange) of varying overhang length (–6 to +10 nt with respect to the chol-DNA strand). L denotes a DNA ladder. (**B–E**) Instantaneous snapshots of all-atom MD simulations of the DNA duplex with one cholesterol tag with either no overhang (B and C) or a 6 nt overhang (D and E), each shown at the beginning (0 μs) and end (0.5 μs) of the equilibrium MD simulation. Close-ups illustrate that cholesterol is more freely accessible without the overhang (C) but shielded by the ssDNA if the overhang is present (E) (Supplementary Movies M2 and M3). (**F**) Cy3-labeled duplex with +6 nt overhang (red) shows attachment to droplet-stabilized giant unilamellar vesicles (brightfield) (61). The cholesterol-shielding overhang does not inhibit lipid membrane interaction. Scale bars denote 50 μm. For images of Atto488-labeled lipids see Supplementary Figure S11.

As applications for cholesterol-modified DNA commonly involve double-stranded DNA, we investigated if aggregation can be controlled through the design of the complementary, unmodified DNA strand utilizing the demonstrated cholesterol–ssDNA interaction. To test this, the clustering chol-DNA strand (seq. b in Figure 1B) was hybridized to complementary strands of various lengths ranging from six nucleotides shorter than the chol-DNA strand (–6) to a ten nucleotide ssDNA overhang adjacent to the cholesterol (+10) (for sequences see Supplementary Table S2). PAGE results in Figure 2A show that although complementary strands without overhang (–6 and 0) prevent complete aggregation (by inhibiting multimerization), transient cholesterol-mediated interactions still remain, indicated by smeared bands. However, the presence of ssDNA overhangs eliminates these transient interactions. A 6 nt overhang on the complementary strand (+6) is sufficient to obtain a clear band in the gel, successfully suppressing both aggregation and transient interactions. The same aggregation prevention was achieved for tocopherol-modified DNA in a separate experiment (Supplementary Figure S9).

The interaction of the cholesterol with the DNA duplex was again investigated by 0.5 μs long all-atom MD simulations. Without overhang, the cholesterol remains freely accessible as there is no ssDNA present to wrap around it (Figure 2B and C). This accessibility of the cholesterol is likely to mediate molecular interactions between modified duplexes causing the observed smeared gel bands. In contrast, the simulation of the cholesterol-modified duplex with a 6 nt overhang shows that the cholesterol is enwrapped by the ssDNA (Figure 2D and E). An independent repeat for each simulation yielded similar results (Supplementary Figure S10 and Supplementary Movies M2 and M3). The combined results of PAGE and simulations show that free cholesterol modifications are more prone to clustering than ones shielded by adjacent ssDNA.

As the purpose of modifying DNA with cholesterol is the interaction of DNA with lipid bilayers, we needed to ensure that the overhang does not impede membrane binding. We therefore incubated artificial, giant unilamellar vesicles (GUVs) with a Cy3-labeled, cholesterol-modified duplex containing a 6 nt overhang. Imaging the vesicles with confocal fluorescence microscopy showed bright fluorescent rings, co-localizing with the lipid membranes, thereby showing successful insertion of the cholesterol into the membrane (Figure 2F). This demonstrates that the presence of the overhang does not inhibit membrane binding. This could be expected as previous studies using hydrophobic porphyrin tags located internally within DNA duplexes similarly showed successful membrane anchoring (63,64).

From these experiments and simulations, we can conclude that the extension of the complementary DNA strand adjacent to the cholesterol moiety can be used as a strategy to avoid aggregation of cholesterol-tagged DNA duplexes.

### DNA nanostructure design and assembly

**Figure 3.**
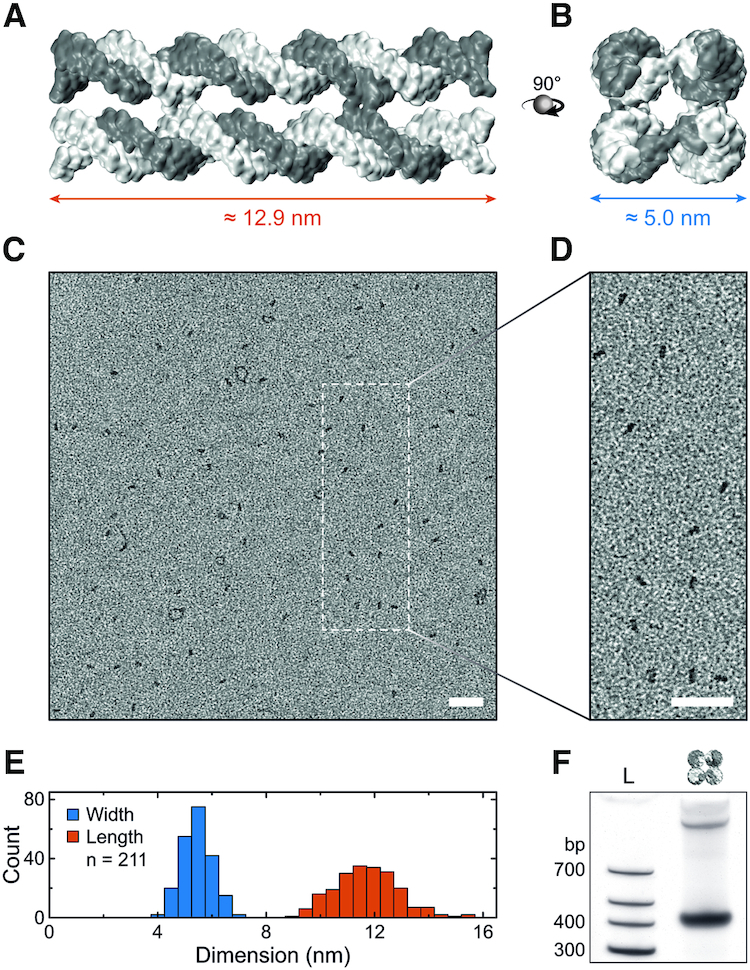
DNA nanostructure design and assembly. (**A**) Side and (**B**) top view of a 3D representation of the DNA nanostructure. Dimensions assume a base pair distance of 0.34 nm, dsDNA diameter of 2.25 nm (65) and interhelical spacing of 2.7 nm (66). (**C**) TEM image of positively stained DNA constructs with a scaled-up view shown in (**D**). Scale bars denote 50 nm. (**E**) Distribution of length and width, as defined in (A) and (B), obtained from TEM images yielding a length of 11.7 ± 1.2 nm and width of 5.4 ± 0.6 nm (mean ± s.d.). (**F**) PAGE of DNA nanostructures showing an intense monomer band and a faint dimer band at shorter run length.

After studying cholesterol-modified ssDNA and dsDNA, we investigated the cholesterol-mediated aggregation of DNA nanostructures. A model DNA nanostructure composed of eight DNA single strands was designed that, in the presence of magnesium and upon thermal annealing, self-assemble into four interconnected DNA duplexes (Figure 3A and B; for sequences see Supplementary Figure S12 and Supplementary Table S3) (20). Cross-overs linking the duplexes are located where DNA backbones are in close proximity, minimizing torsional stress (67). Stacking interactions between blunt double-stranded ends were most efficiently prevented by ssDNA extensions composed of three thymine nucleotides on both ends of the design (Supplementary Figure S13) (68–70).

Successful assembly of the DNA nanostructures was verified with transmission electron microscopy (TEM, Figure 3C). The magnified view in Figure 3D illustrates that the majority of structures are of rectangular geometry consistent with monomeric nanostructures lying flat on the TEM grid. The lengths and widths of over 200 monomeric DNA nanostructures were measured from eight images comparable to the overview shown in Figure 3C (Supplementary Figure S14). The determined average length of 11.7 ± 1.2 nm and width of 5.4 ± 0.6 nm (mean ± s.d.) agree very well with the expected dimensions (Figure 3E). Reduced staining of the ssDNA extensions compared to the 10.9 nm dsDNA core could explain the slightly shorter length while uranyl atoms predominantly binding to the DNA backbone (71) could explain the slightly larger width.

Bulk analysis of assembled DNA nanostructures with PAGE shows an intense band suggesting a high yield of ≈83% of monomeric structures (Figure 3F), as observed in the TEM images. A low-intensity band at shorter run length indicates ≈17% of dimeric structures. The combined results of gel electrophoresis and TEM demonstrate successful assembly of the unmodified DNA nanostructures with the expected dimensions and geometry.

### DNA nanostructure aggregation dependence on cholesterol number, linker type, and modification position

**Figure 4.**
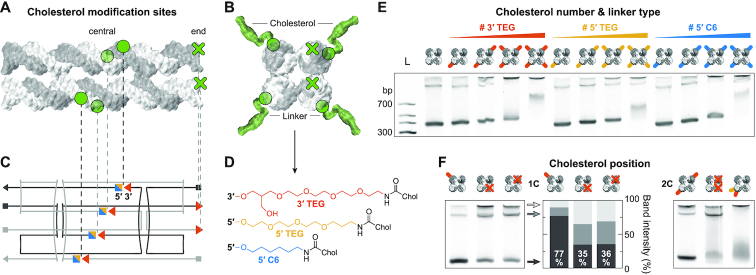
DNA nanostructure aggregation dependence on cholesterol number, linker type, and modification position. (**A**) Side and (**B**) top view of the DNA nanostructure with cholesterol modification sites (green) located at the center (circle) or the end (cross) of the construct. (**C**) 2D design scheme illustrating the DNA single strand pathways. (**D**) Chemical structures of three different cholesterol–DNA linkers with possible modification positions shown in (C). (**E**) Comparison of DNA nanostructures assembled with 1 to 4 cholesterol groups (indicated by colored lines) for each linker type. (**F**) PAGE illustrating the influence of the cholesterol modification position on the aggregation of DNA nanostructures. For DNA sequences see Supplementary Tables S4 to S6.

A key feature of the design is an array of 3′ and 5′ ends in the central region of the DNA nanostructure, allowing for the specific positioning of cholesterol modifications (Figure 4A–C). They are strategically placed considering the DNA’s helical pitch such that cholesterol groups face diagonally away from the structure, aiding their accessibility after assembly (Figure 4B). This facilitates the systematic analysis of the cholesterol-mediated interaction between the DNA nanostructures regarding cholesterol number, linker type, and modification position.

First, DNA nanostructures have been assembled with 1 to 4 cholesterol modifications covalently bound to the DNA using three different commercially available linkers: 3′ and 5′ end TEG-linkers, and a 5′ hexane (C6) linker (Figure 4D). They differ in length, modification position (3′ or 5′), and hydrophobicity with TEG being less hydrophobic than the C6 chain due to the additional oxygen atoms. PAGE analysis reveals stepwise band shifts to shorter run lengths with each additional cholesterol group for all linker types (Figure 4E). This can be explained by the increasing hydrodynamic radius of the nanostructures for each additional cholesterol tag. For 1 to 3 cholesterol groups, monomer bands remain relatively sharp, however, aggregation in the gel wells gradually increases. All structures with 4 cholesterol modifications show a smeared, less intense band with a band shift much larger than the previously observed consistent shifts for each cholesterol addition. This suggests that cholesterol-mediated interactions are now inducing significant aggregation of the DNA nanostructures. Note that to establish a homogeneous attachment chemistry, and in agreement with our previous work (20), a single adenine nucleotide was included as a spacer preceding each central cholesterol modification. However, this spacer did not significantly influence the observed cholesterol-mediated aggregation characteristics (Supplementary Figure S15). Next, the influence of the cholesterol modification position was studied. Figure 4F shows the PAGE analysis of DNA nanostructures folded with 1 or 2 TEG-linked cholesterol groups (1C and 2C, respectively). While a single cholesterol tag in the central region shifts the monomer band, it does not change the band intensity nor induce aggregation. However, introducing a single cholesterol at the end of the design reduces the monomer band intensity to ≈35% (compared to 77% if on the side), while dimer and multimer intensities increased by 19% and 23%, respectively. Similar cholesterol-mediated dimerization has been reported on an alternative DNA cube construct (35). Our results suggest aggregation of the nanostructures similar to the one driven by base-stacking interactions (Supplementary Figure S13).

Two central cholesterol modifications at opposing sides also yield a sharp monomer band (Figure 4F). This contrasts with two cholesterol tags placed at the end of the structure, causing a smeared monomer band with reduced intensity, while the intensity of the dimer band increases. Furthermore, placing two cholesterol tags right next to each other at the same location results in a significantly smeared monomer band. These results suggest an aggregation dependence not only on the number of cholesterol tags but also the modification position.

### Controlling cholesterol-mediated DNA nanostructure aggregation by ssDNA overhangs

**Figure 5.**
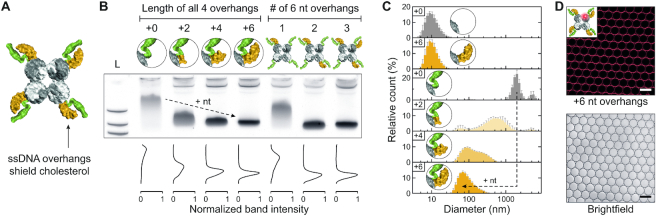
Controlling cholesterol-mediated DNA nanostructure aggregation by ssDNA overhangs. (**A**) Top view of a 3D model of the DNA nanostructure including four cholesterol modifications (green), each shielded by a 6 nt overhang (orange). (**B**) PAGE showing reduced aggregation of 4C DNA nanostructures assembled with either increasing ssDNA overhang length (+2 to +6 nt) or increasing number of 6 nt overhangs adjacent to the cholesterol. Band intensity graphs were normalized to the +6 nt band as it exhibited the highest intensity. (**C**) DLS analysis of DNA nanostructures without and with four cholesterol tags demonstrating reduced aggregation if +2, +4 or +6 nt overhangs are employed. Histograms represent the average volume distribution values of five measurements (error bars denote s.d.). (**D**) Confocal microscopy images of Cy3-labeled DNA nanostructures (red) with four cholesterol tags, each shielded by a 6 nt overhang, showing attachment to lipid membranes (brightfield). Scale bars denote 50 μm. See Supplementary Figure S11 for images of Atto488-labeled lipids and Supplementary Table S7 for DNA sequences.

Having studied the cholesterol-mediated aggregation of the DNA nanostructure, we then applied our mechanism of preventing aggregation by shielding the cholesterol modifications with adjacent ssDNA overhangs (Figure 5A). For this purpose, we extended the 5′ ends in the central region of the design by 2, 4 or 6 thymine nucleotides to obtain ssDNA overhangs adjacent to the cholesterol tags modified to the central 3′ ends (compare Figure 4C). As four central cholesterol modifications consistently led to aggregation, we assembled DNA nanostructures with four cholesterol tags without and with 2, 4 or 6 nt overhangs adjacent to all cholesterol groups. Our earlier results suggest that overhangs can successfully prevent aggregation. As expected, structures without ssDNA overhangs again demonstrated clustering in PAGE. However, with increasing overhang lengths the initially smeared band gradually shifts to longer run lengths while increasing in sharpness and intensity (Figure 5B). This behavior signifies a gradually decreasing hydrodynamic radius as well as increasing structural homogeneity, both indicative of successful aggregation suppression. An overhang length of 6 nt was sufficient to achieve a sharp monomer band, consistent with the results of the DNA duplex studies (compare Figure 2A). Structures with 6 nt overhangs also produced consistent band shifts for each additional cholesterol without significant changes to the intensity of the monomer band (Supplementary Figure S16). Moreover, varying the number of 6 nt overhangs showed that while one overhang was insufficient, already from two overhangs onwards a significantly sharper monomer band was observed suggesting successful aggregation prevention (Figure 5B). We furthermore successfully reduced aggregation of an additional cholesterol-modified DNA nanostructure design, confirming the versatility of our method across different constructs (Supplementary Figure S17).

We performed DLS measurements to show that the observed gel electrophoresis results are representative for the aggregation behavior of the DNA constructs in solution. While DNA nanostructures without cholesterol groups showed no aggregation, modification with four cholesterol tags led to significant clustering, with diameters of the aggregates exceeding 1000 nm (Figure 5C). Upon addition of increasingly longer ssDNA overhangs, aggregation is gradually reduced, consistent with the PAGE experiments. DLS results confirm that the effects from non-denaturing PAGE are representative for the cholesterol-mediated aggregation behavior of DNA nanostructures in solution.

Lastly, we verified that DNA nanostructures with 4 cholesterol tags, each shielded by adjacent 6 nt overhangs, still bind to lipid bilayers. Analogous to experiments performed with the shielded DNA duplex, confocal microscopy of droplet-stabilized GUVs containing Cy3-labeled DNA nanostructures revealed that membrane binding was not inhibited by the ssDNA overhangs (Figure 5D).

## DISCUSSION

Our detailed analysis of cholesterol-modified ssDNA, dsDNA and DNA nanostructures shows that it is possible to control the aggregation of cholesterol-modified DNA nanostructures following a few simple design rules.

For cholesterol-modified ssDNA, our results demonstrate a sequence-dependent aggregation behavior independent of the production batch, supplier, or storage conditions. Sequences prone to forming dimers or multimers generally showed significant aggregation and should be avoided by optimizing the sequences using structure prediction tools such as NUPACK. Terminal thymine nucleotides generally avoided aggregation best, thus we recommend incorporating a poly-T section to precede the cholesterol moiety if the DNA sequence can be chosen freely.

Our atomistic MD simulations of poly-T chol-DNA showed interactions of the hydrophobic nucleobases with the hydrophobic cholesterol moiety causing the ssDNA to wrap around the cholesterol group. We postulate that this prevents hydrophobic interactions with other strands in solution, consistent with our experimental observations.

For mixed DNA sequences, the behavior is more complex. It has been shown that cholesterol and its esters can form stable complexes with DNA duplexes (72). Furthermore, inter-cholesterol hydrophobic interactions have been demonstrated to increase the melting temperature of nucleic acid duplexes and triplexes by up to 13°C and 30°C, respectively (73). Such interactions could stabilize secondary structures by raising their melting temperature thereby inducing the observed sequence-dependent aggregation of chol-DNA. Consequently, experiments involving chol-DNA require careful consideration of the DNA sequence.

We further demonstrated that the incorporation of cholesterol tags into DNA nanostructures can be successfully analyzed with non-denaturing PAGE with a visible shift for each additional cholesterol group. While nanostructure clustering does not significantly depend on the DNA–cholesterol linker type, it is the number and location of the hydrophobic tags that proved to be the governing factors. The fact that DNA nanostructures with the same number of cholesterol tags can behave significantly different in native PAGE suggests that interactions between the nanostructures, rather than with the polyacrylamide gel matrix, are governing. This is further supported by the DLS measurements performed in solution. As we furthermore showed that denaturing conditions mask the cholesterol-mediated aggregation, studying interactions between hydrophobically modified DNA nanostructures requires non-denaturing gel electrophoresis to accurately judge and precisely adjust aggregation of designs with a higher number of cholesterol tags.

Most importantly, we demonstrated that the hydrophobic interactions of cholesterol-tagged DNA can be easily controlled by incorporating an ssDNA overhang adjacent to the hydrophobic group. In agreement with the MD simulations, the ssDNA overhang most likely presents a steric hindrance, effectively preventing cholesterol-mediated aggregation. Due to the flexible nature of the ssDNA overhang, it does not impede insertion of the cholesterol-tagged DNA into a lipid bilayer membrane as we showed with confocal fluorescence imaging. Adjusting the length of the overhang can be utilized to precisely tune the degree of hydrophobic interaction. This allows for controlling cholesterol interactions simply by the rational design of the ssDNA overhang. The method also avoids the necessity of multi-unit membrane incorporation strategies and the use of surfactants, thereby circumventing the drawbacks of these strategies. Furthermore, it enables to increase the number of cholesterol modifications per DNA nanostructure, thereby facilitating enhanced membrane interaction.

In summary, to effectively prevent cholesterol-mediated aggregation, we advise to (1) employ DNA sequences with minimal secondary structure formation, (2) avoid guanine nucleotides preceding the cholesterol tag, but rather include a terminal poly-T section, and (3) to incorporate ssDNA overhangs (e.g. 6T) adjacent to the cholesterol group when designing DNA duplexes and nanostructures such as DNA origami.

The results of our study present a guide to control and prevent aggregation of cholesterol-modified ssDNA, dsDNA and DNA nanostructures using simple design rules and no additional components. Our novel, cost-efficient, and easy-to-implement strategy to control aggregation of cholesterol-modified DNA nanostructures can readily be applied to existing designs without significant modifications. Additionally, it is not limited to cholesterol but can be applied to other hydrophobic modifications as already demonstrated for tocopherol. Facilitating greater control in interfacing DNA nanostructures with lipid bilayers and cell membranes will lead to powerful DNA nanosystems with applications in research, biomedicine and beyond.

## DATA AVAILABILITY

The authors declare that all data supporting the findings of this study are available within the paper (and its Supplementary Data).

## Supplementary Material

gkz914_Supplemental_Files
