# Supplementary material for: Controlling aggregation of cholesterol-modified DNA nanostructures
Source: Nucleic Acids Res. 2019 Oct 23;47(21):11441–51. doi: 10.1093/nar/gkz914 (PMC6868430; doi:10.1093/nar/gkz914)
Supplement: gkz914_Supplemental_Files [file gkz914_supplemental_files.zip › 190927_NAR_CholDNA_Supp_revised_v2.docx]

**Supplementary Data**

**Controlling aggregation of cholesterol-modified DNA nanostructures**

Alexander Ohmann^1^, Kerstin Göpfrich^1,2^, Himanshu Joshi^3^, Rebecca F. Thompson^4^, Diana Sobota^1^, Neil A. Ranson^4^, Aleksei Aksimentiev^5^ and Ulrich F. Keyser^1,^*

^1^ Cavendish Laboratory, University of Cambridge, JJ Thomson Avenue, Cambridge CB3 0HE, United Kingdom.

^2^ Max Planck Institute for Medical Research, Department of Cellular Biophysics, Jahnstraße 29, 69120, Heidelberg, Germany.

^3^ Department of Physics, University of Illinois at Urbana-Champaign, 1110 West Green Street, Urbana, IL 61801 USA.

^4^ Faculty of Biological Sciences, University of Leeds, Leeds LS2 9JT, United Kingdom.

^5^ Department of Physics and Beckman Institute for Advanced Science and Technology, University of Illinois at Urbana-Champaign, 1110 West Green Street, Urbana, IL 61801, USA.

**Supplementary Table S1.** Sequences of DNA strands used in PAGE experiments shown in main text Figure 1. Regarding the batches of DNA sequences analyzed in main text Figure 1B: batch 1 was ordered on 28.10.2014, batch 2 on 29.01.2015, batch 3 on 29.04.2015.

| **Panel** | **DNA strand** | **Sequence (5’ to 3’)** |
| --- | --- | --- |
| 1B | 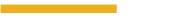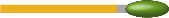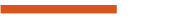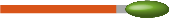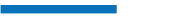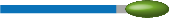 a | AAAACGCTAAGCCACCTTTAGATCCAAA |
|  |  | AAAACGCTAAGCCACCTTTAGATCCAAA/3CholTEG/ |
|  | b | AAACTCCCGGAGTCCGCTGCTGATCAAA |
|  |  | AAACTCCCGGAGTCCGCTGCTGATCAAA/3CholTEG/ |
|  | c | GGATCTAAAGGACTTCTATCAAAGACGGGACGACTCCGGGAG |
|  |  | GGATCTAAAGGACTTCTATCAAAGACGGGACGACTCCGGGAG/3CholTEG/ |
| 1C | 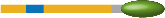 | AAACTCCC**G**GAGTCCGCTGCTGATCAAA/3CholTEG/ |
|  | 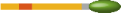 | AAACTCCC**A**GAGTCCGCTGCTGATCAAA/3CholTEG/ |
| 1D | 20T | TTTTTTTTTTTTTTTTTTTT |
|  | 30T | TTTTTTTTTTTTTTTTTTTTTTTTTTTTTT |
|  | 40T | TTTTTTTTTTTTTTTTTTTTTTTTTTTTTTTTTTTTTTTT |
|  | 50T | TTTTTTTTTTTTTTTTTTTTTTTTTTTTTTTTTTTTTTTTTTTTTTTTTT |
|  | 20T-chol | TTTTTTTTTTTTTTTTTTTT/3CholTEG/ |
|  | 30T-chol | TTTTTTTTTTTTTTTTTTTTTTTTTTTTTT/3CholTEG/ |
|  | 40T-chol | TTTTTTTTTTTTTTTTTTTTTTTTTTTTTTTTTTTTTTTT/3CholTEG/ |
|  | 50T-chol | TTTTTTTTTTTTTTTTTTTTTTTTTTTTTTTTTTTTTTTTTTTTTTTTTT/3CholTEG/ |
| 1F | 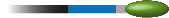 | TTTTTTTTTTTTTTTTTTTTTTTTTTT**TTT** |
|  | 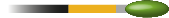 | TTTTTTTTTTTTTTTTTTTTTTTTTTT**CCC** |
|  | 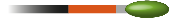 | TTTTTTTTTTTTTTTTTTTTTTTTTTT**GGG** |
|  | 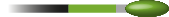 | TTTTTTTTTTTTTTTTTTTTTTTTTTT**AAA** |
| 1G | 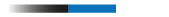 | ACTGTCGAACATTTTTTGCCATAATA**TAT** |
|  | 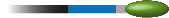 | ACTGTCGAACATTTTTTGCCATAATA**TAT**/3CholTEG/ |
|  | 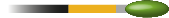 | ACTGTCGAACATTTTTTGCCATAATA**GCG**/3CholTEG/ |
|  | 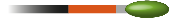 | ACTGTCGAACATTTTTTGCCATAATA**AAA**/3CholTEG/ |


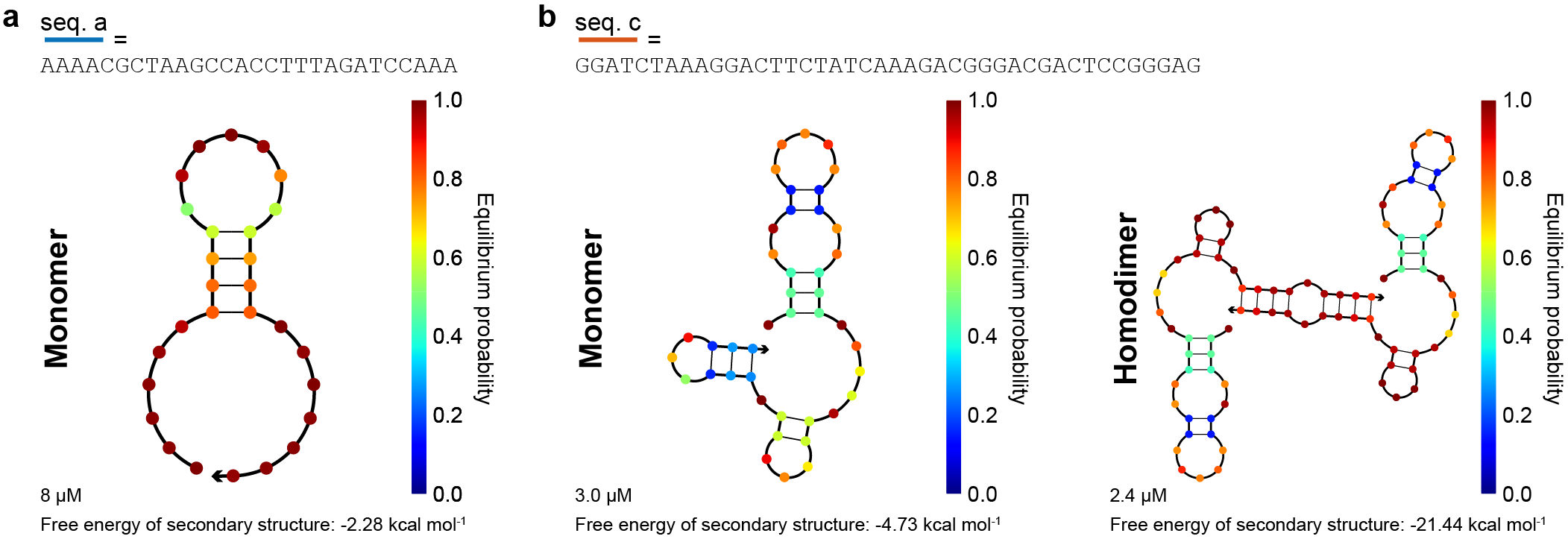


**Supplementary Figure S1.** NUPACK minimum free energy calculations of secondary structures of sequence a (**a**) and sequence c (**b**) analyzed in main text Figure 1B. Calculations were performed at a temperature of 18 °C and a DNA concentration of 8 µM with 50 mM NaCl and 11 mM MgCl_2_. Sequence a showed no dimerization at all. Except for the additional NaCl, these parameters match the experimental conditions chol-DNA strands were subjected to during PAGE.


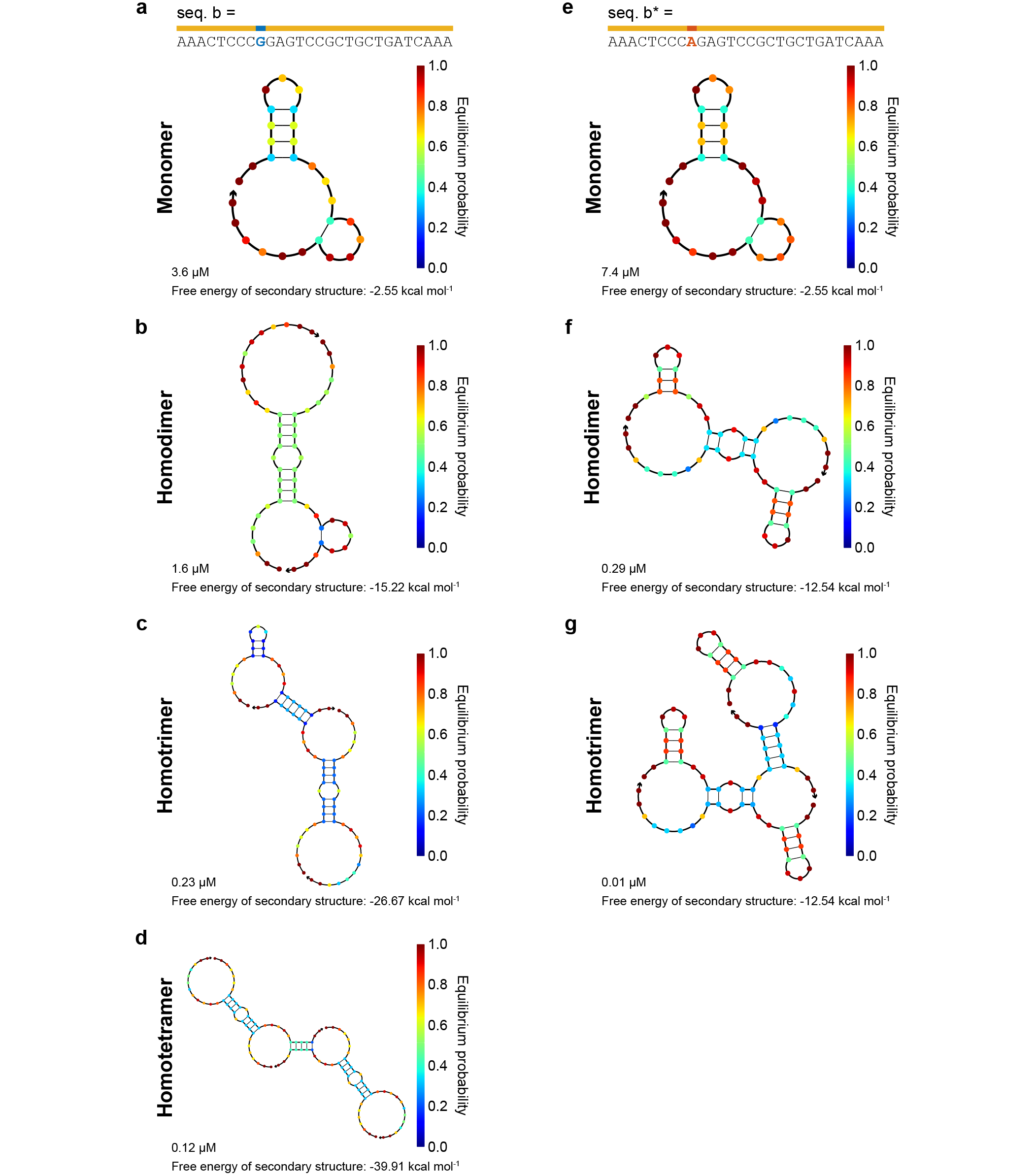


**Supplementary Figure S2.** NUPACK minimum free energy calculations of secondary structures of DNA sequences analyzed in main text Figure 1B and C with either a guanine (sequence b, **a**-**d**) or an adenine (sequence b*, **e**-**g**) as the ninth base. Calculations were performed at a temperature of 18 °C and a DNA concentration of 8 µM with 50 mM NaCl and 11 mM MgCl_2_.


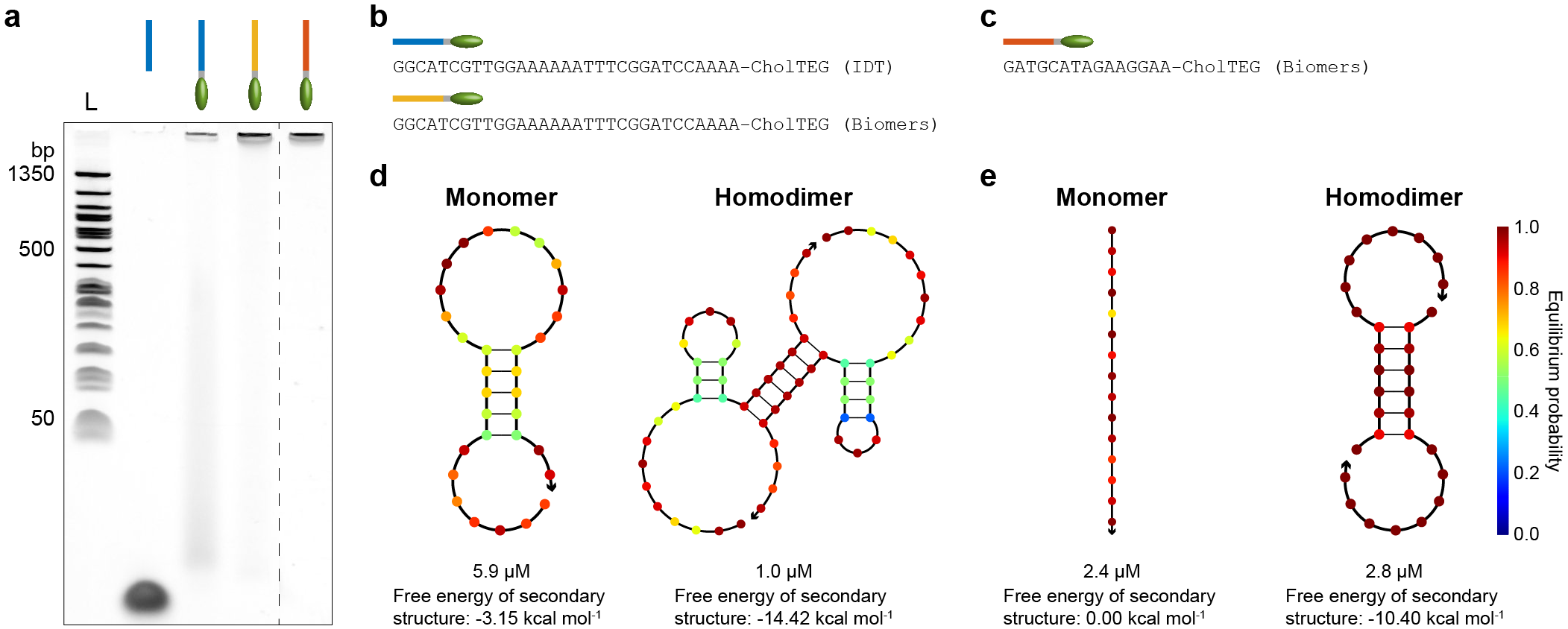


**Supplementary Figure S3.** Further examples of cholesterol-modified ssDNA sequences that demonstrate clustering in the gel well. **a** PAGE image of one unmodified and three cholesterol-modified ssDNA strands with their sequences and supplier shown in (**b**) and (**c**). Samples were run on the same gel but space between lanes was omitted as indicated by the dashed line. L denotes a 50 bp DNA ladder (New England Biolabs). **d**,**e** NUPACK free energy calculations of the analyzed DNA sequences shown in (**b**) and (**c**), respectively, illustrating a tendency for homodimerization. Calculations were performed at a temperature of 18 °C and a DNA concentration of 8 µM with 50 mM NaCl and 11 mM MgCl_2_.


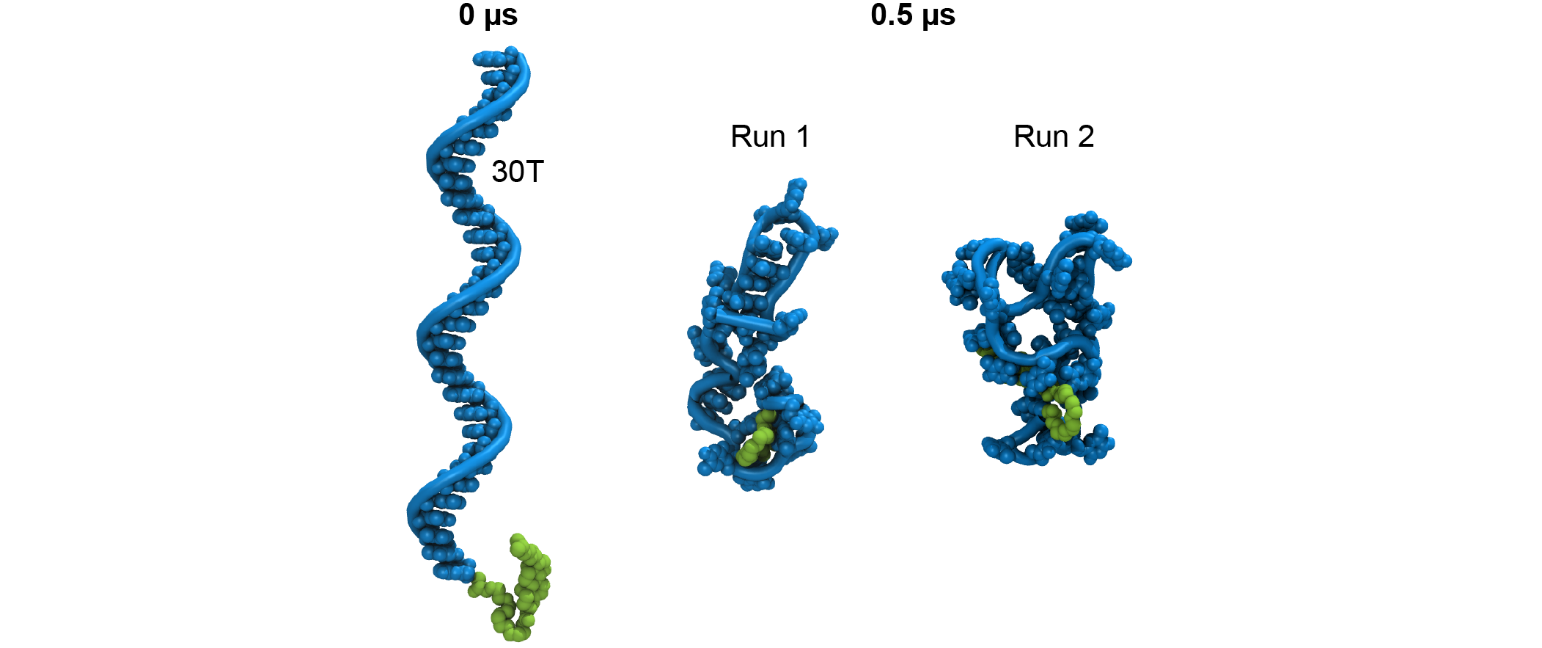


**Supplementary Figure S4.** Instantaneous snapshots of all-atom MD simulation of cholesterol-modified 30 thymidine ssDNA (30T) shown at the beginning (0 µs) and end (0.5 µs) of two independent simulation runs starting from the same initial configuration. The ssDNA wraps around the cholesterol group throughout both simulation runs. Water and ions are not shown for clarity. For simulation movies see Supplementary Movie M1.


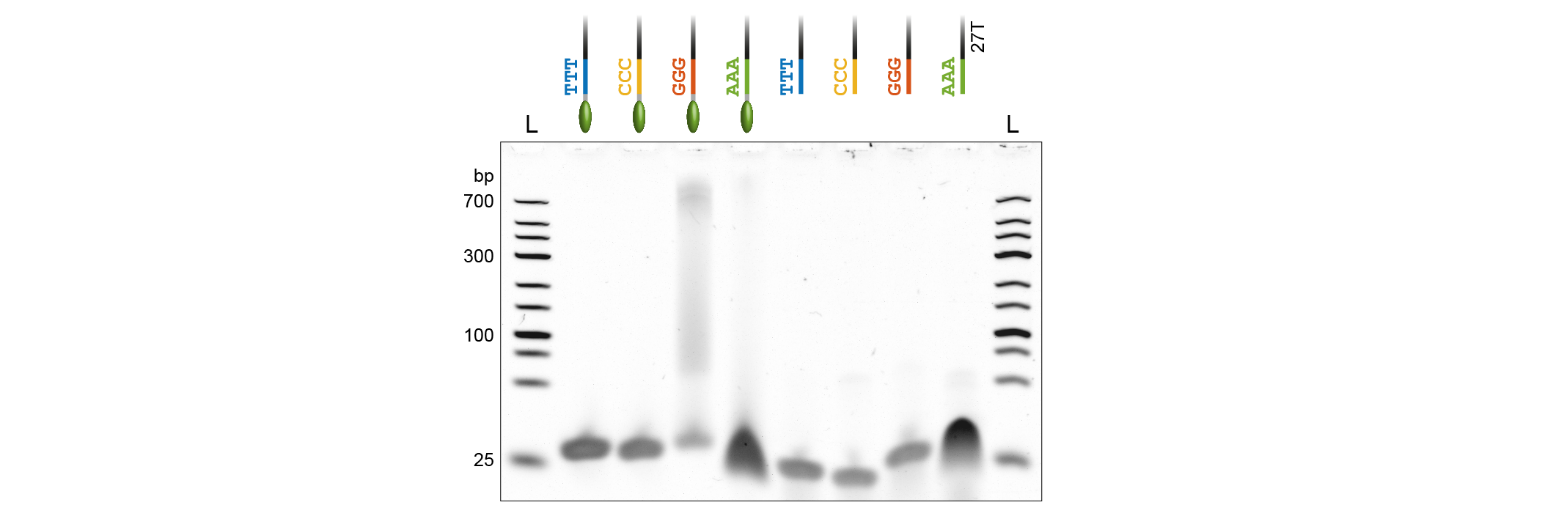


**Supplementary Figure S5.** Extended view of gel image shown in main text Figure 1F comparing the effects of different terminal bases on cholesterol-modified and unmodified DNA single strands.


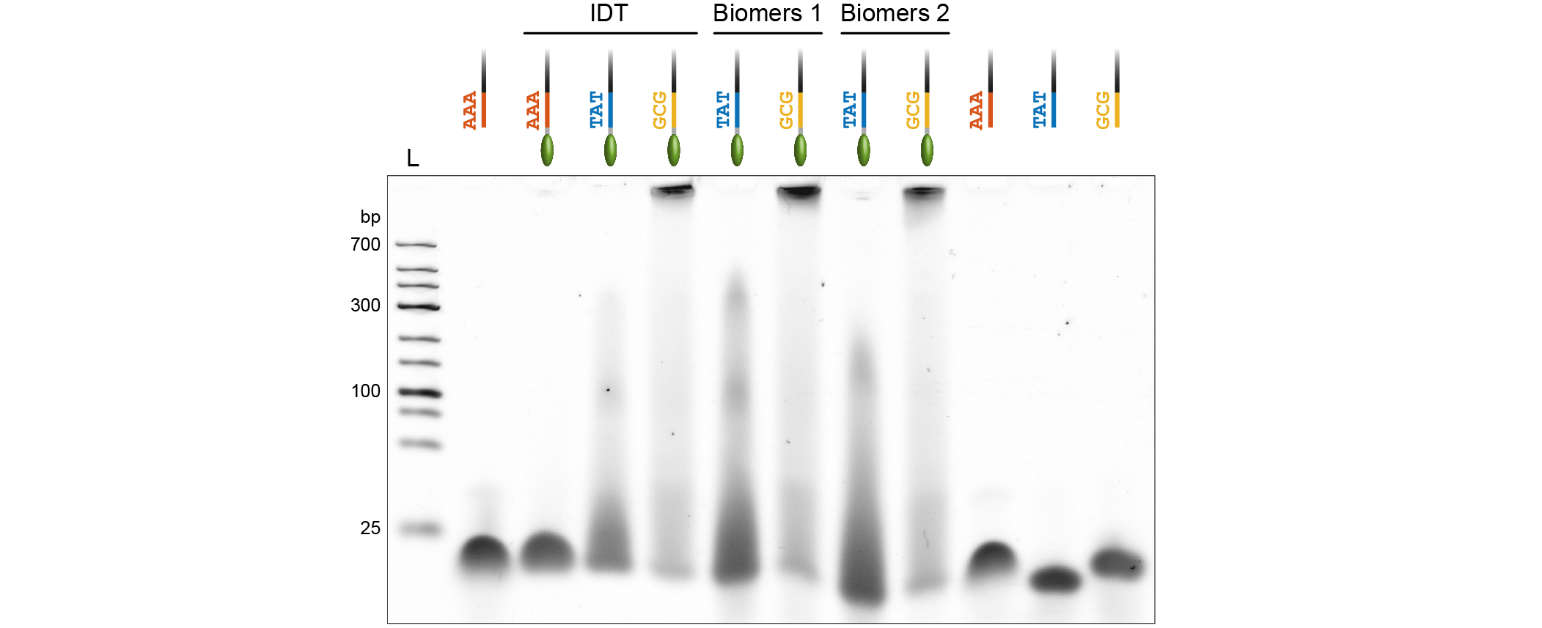


**Supplementary Figure S6.** PAGE comparing chol-DNA strands with varying terminal bases regarding supplier and storage conditions. Chol-DNA strands from IDT were acquired and diluted in Milli-Q water less than three weeks before the experiment. Chol-DNA strands from biomers.net were acquired dry in two aliquots from the same production batch for each DNA sequence. The “Biomers 1” aliquots were dissolved to 100 µM in Milli-Q water 3 years and 11 months prior the experiment. “Biomers 2” aliquots were stored dry at -20 °C and only dissolved two months prior the experiment. Once diluted in water, all chol-DNA strands were stored at 4 °C. The sequence of the DNA strand was ACTGTCGAACATTTTTTGCCATAATA (seq. d, black part in the figure) followed by three additional nucleotides at the 3’ end, as indicated.


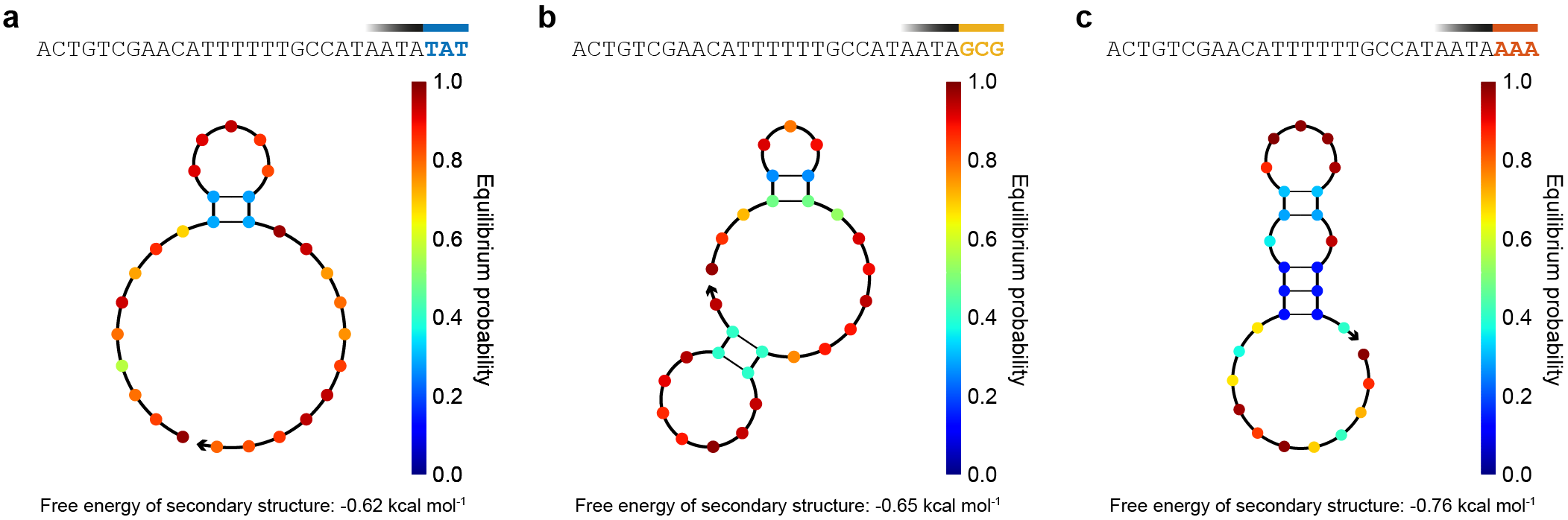


**Supplementary Figure S7.** NUPACK minimum free energy calculations of secondary structures of DNA sequences analyzed in main text Figure 1G terminating either with TAT (**a**), GCG (**b**), or AAA (**c**). Calculations were performed at a temperature of 18 °C and a DNA concentration of 8 µM with 50 mM NaCl and 11 mM MgCl_2_. Dimer formation was below 0.7 % for all sequences.


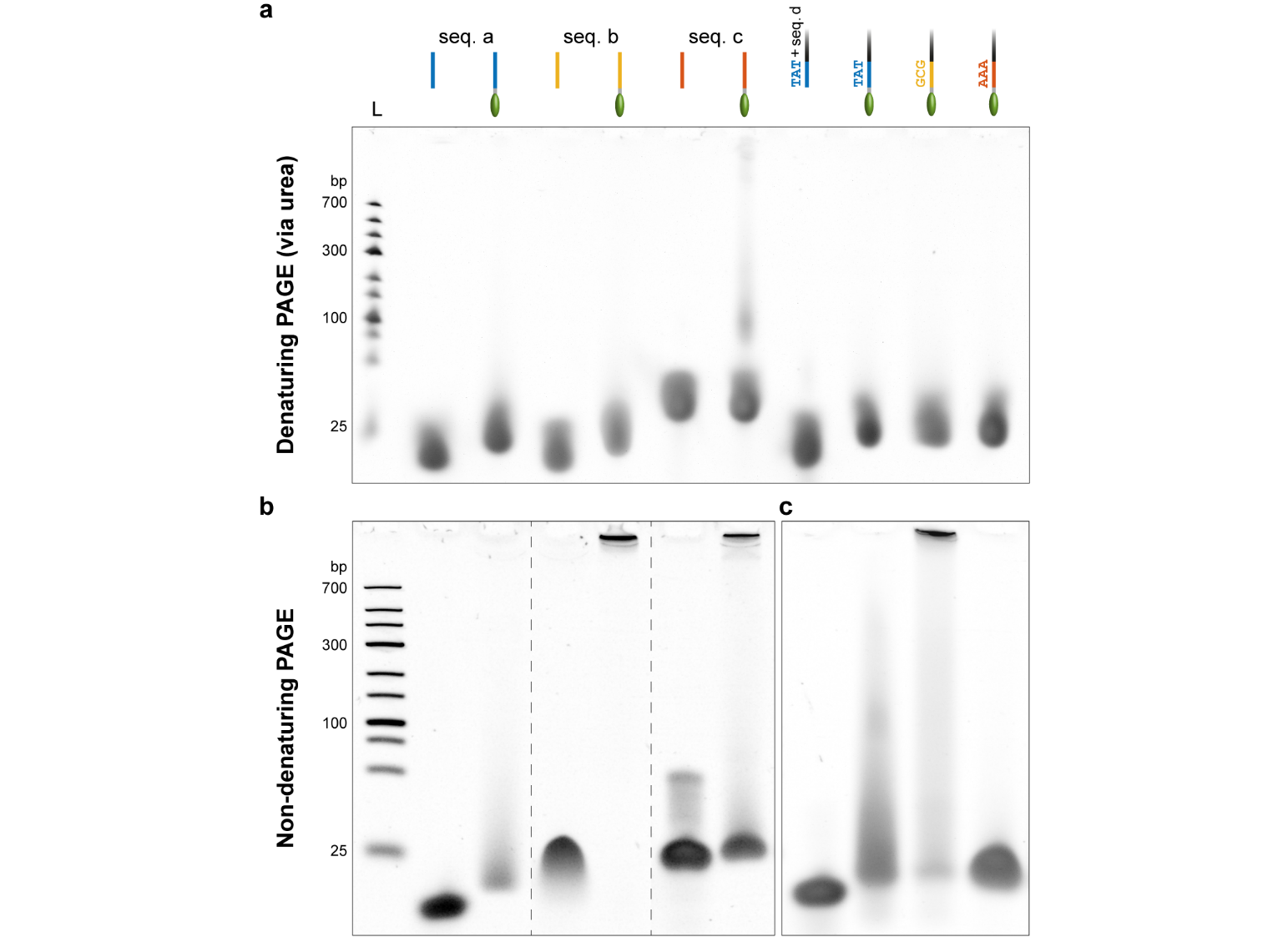


**Supplementary Figure S8.** Analysis of chol-DNA using denaturing and non-denaturing PAGE. **a** Denaturing PAGE of unmodified and cholesterol-modified ssDNA performed under the same conditions as for non-denaturing PAGE (see Materials & Methods), except that 8 M urea was added to the gel mixture. DNA sequences were the same as analyzed in main text Figure 1B (for chol-DNA only batch 1 was used) and Figure 1G (see Supplementary Table S1 for sequences). Denaturing PAGE of the chol-DNA completely conceals any sequence-dependent, cholesterol-mediated aggregation. **b,c** The same DNA sequences as shown in (**a**) but analyzed with non-denaturing PAGE. These gel images are the same as in main text Figure 1B (except that batches 2 and 3 were omitted, indicated by dashed lines) and Figure 1G, but presented again for direct comparison. Differences in band shape between denaturing and non-denaturing gels were caused by the diffusion of urea into the gel wells which prevented the sample from sinking all the way to the bottom of the wells upon loading.

**Supplementary Table S2.** Sequences of DNA strands used for DNA duplex overhang experiments.

| **DNA strand** | **Sequence (5’ to 3’)** | **Supplier** |
| --- | --- | --- |
| ss | AAACTCCCGGAGTCCGCTGCTGATCAAA | IDT |
| ss chol | AAACTCCCGGAGTCCGCTGCTGATCAAA/3CholTEG/ | IDT |
| comp -6 | CAGCAGCGGACTCCGGGAGTTT | IDT |
| comp 0 | TTTGATCAGCAGCGGACTCCGGGAGTTT | IDT |
| comp +2 | CCTTTGATCAGCAGCGGACTCCGGGAGTTT | IDT |
| comp +4 | TACCTTTGATCAGCAGCGGACTCCGGGAGTTT | IDT |
| comp +6 | ACTACCTTTGATCAGCAGCGGACTCCGGGAGTTT | IDT |
| comp +8 | GTACTACCTTTGATCAGCAGCGGACTCCGGGAGTTT | IDT |
| comp +10 | TCGTACTACCTTTGATCAGCAGCGGACTCCGGGAGTTT | IDT |
| comp + 6_Cy3 | ACTACCTTTGATCAGCAGCGGACTCCGGGAGTTT/3Cy3Sp/ | IDT |


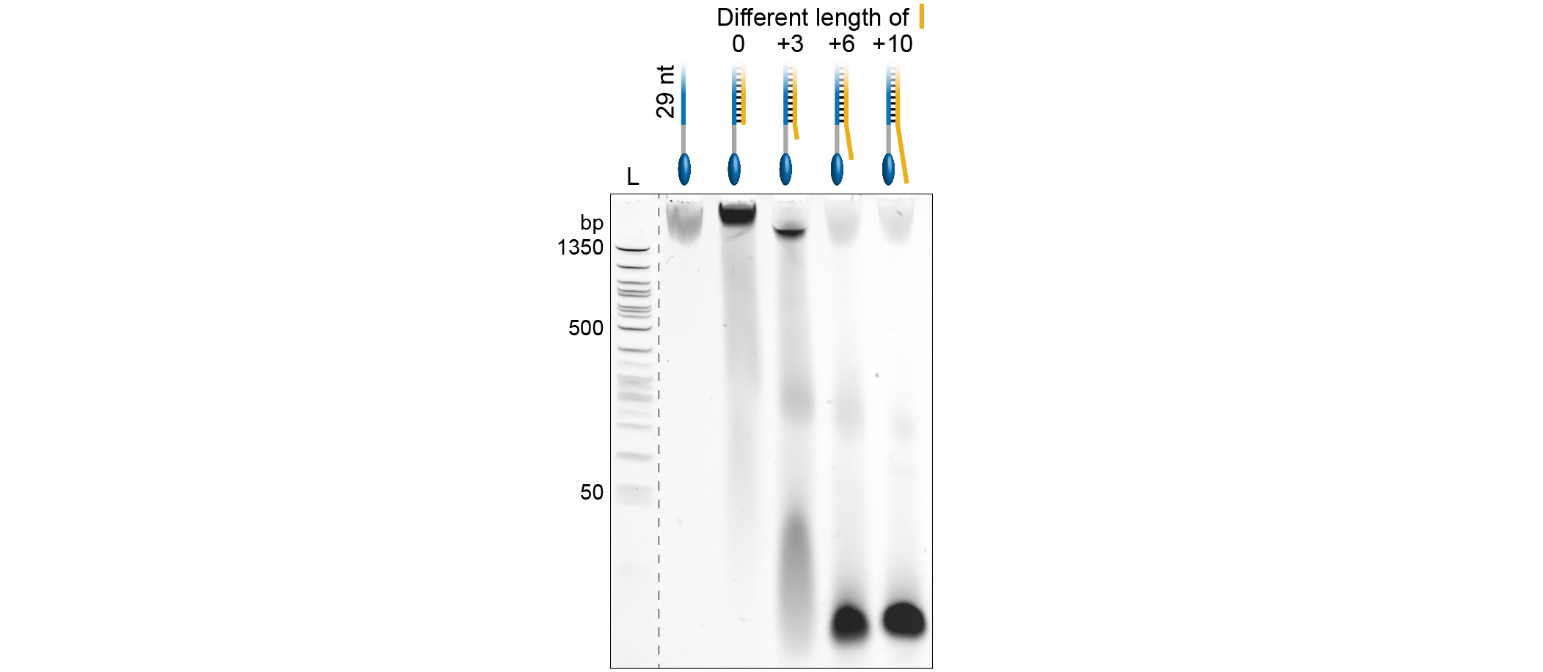


**Supplementary Figure S9.** Preventing aggregation of tocopherol-modified ssDNA by adjacent DNA. 5’ tocopherol-modified ssDNA (Biomers, Sequence: ATATATTATGGCAAAAAATGTTCGACAGT) shows aggregation in PAGE (blue, first lane after the ladder). Aggregation can be successfully suppressed by hybridization to a complementary strand which is extended by six or more nucleotides beyond the length of the tocopherol-modified strand. Sequence of the complementary strand including +10 nt: ACTGTCGAACATTTTTTGCCATAATATATCTGTCACTTG. For shorter strands the sequence was shortened at the 3’ end accordingly. Samples were run on the same gel but space between samples and ladder was omitted as indicated by the dashed line. L denotes a 50 bp DNA ladder (New England Biolabs).


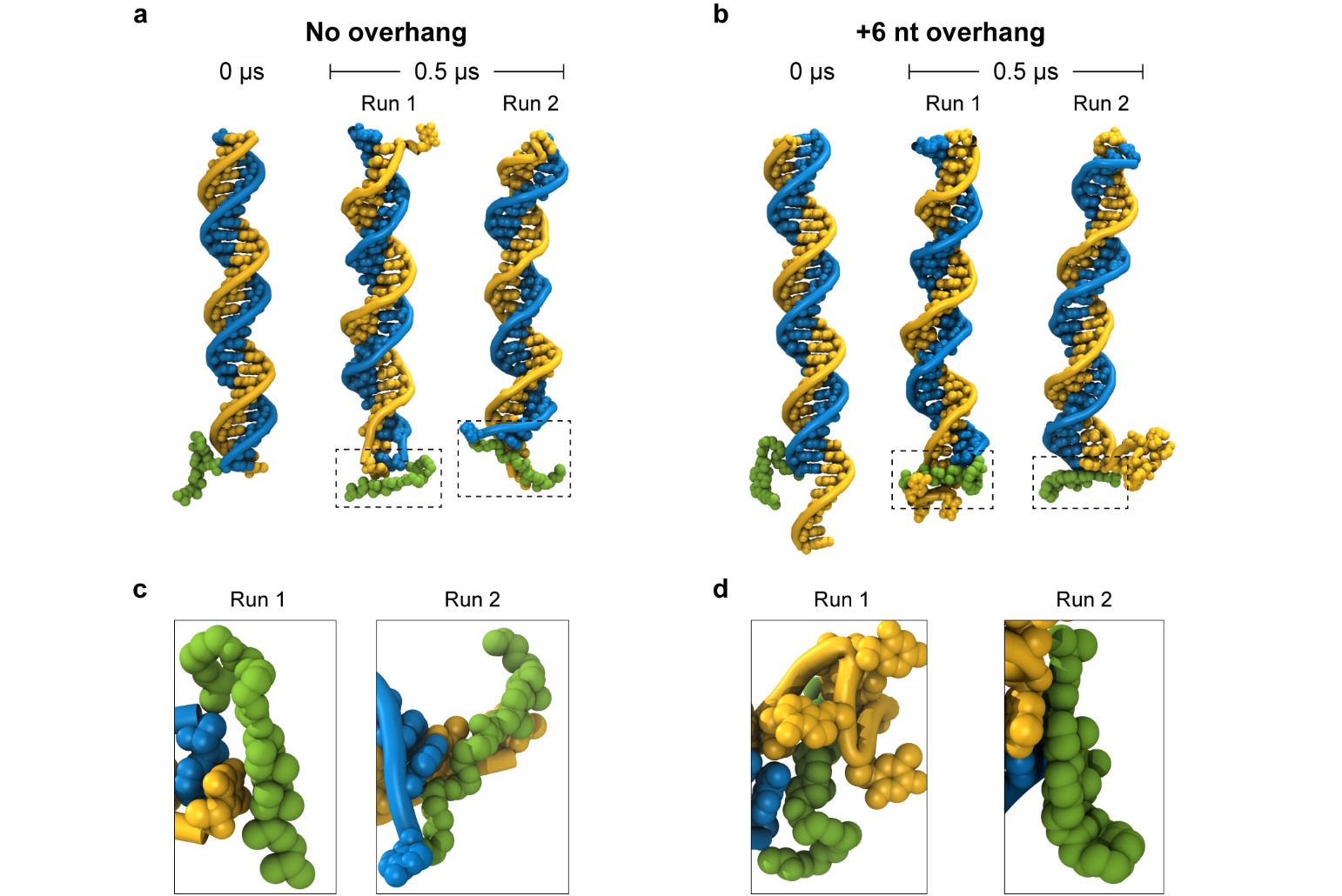


**Supplementary Figure S10.** All-atom MD simulations of DNA duplex with one cholesterol tag either without overhang (**a,c**) or with a 6 nt overhang (**b,d**) shown at the beginning (0 µs) and end (0.5 µs) of the simulations. Two independent simulation runs starting from the same initial configuration were performed for each system.


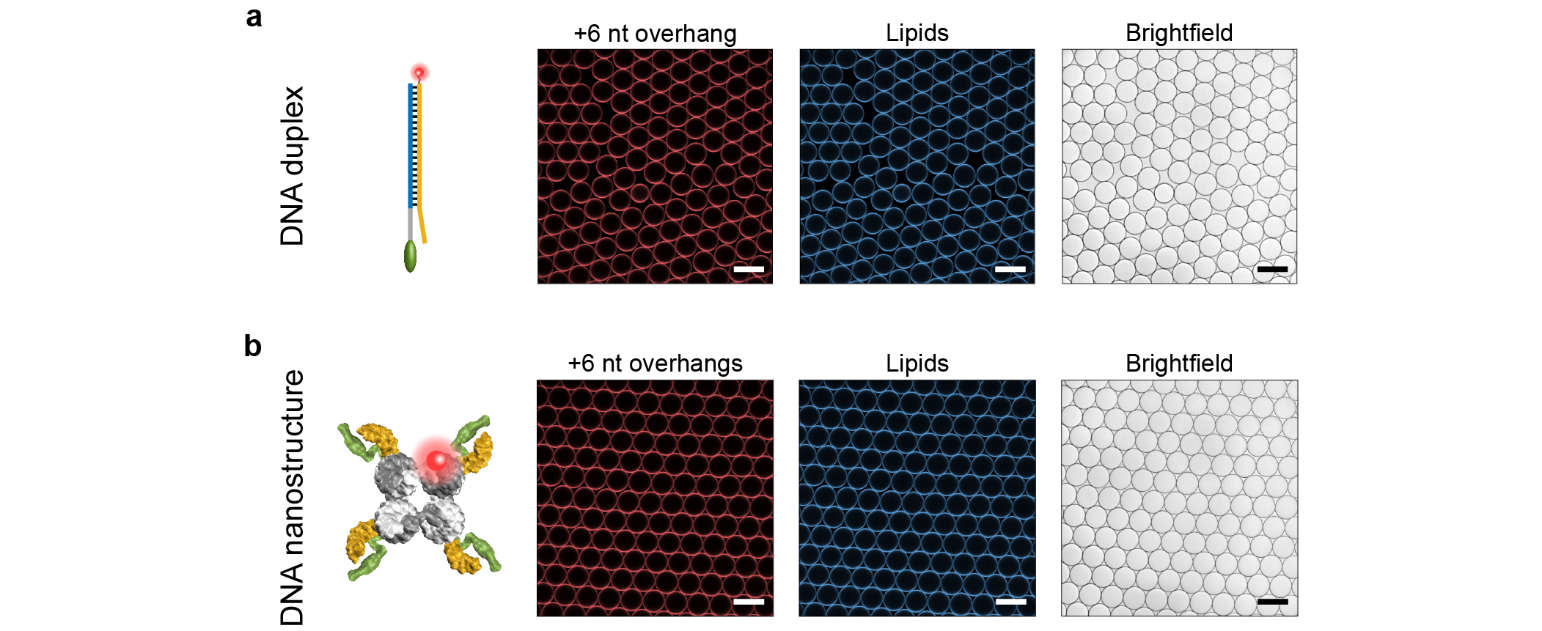


**Supplementary Figure S11.** Confocal microscopy images of Cy3-labeled (red) DNA duplexes (**a**) or DNA nanostructures (**b**) attaching to membranes of lipid vesicles with 0.5 mol% Atto488-labeled lipids (blue) together with brightfield images of the vesicles. Scale bars denote 50 µm.


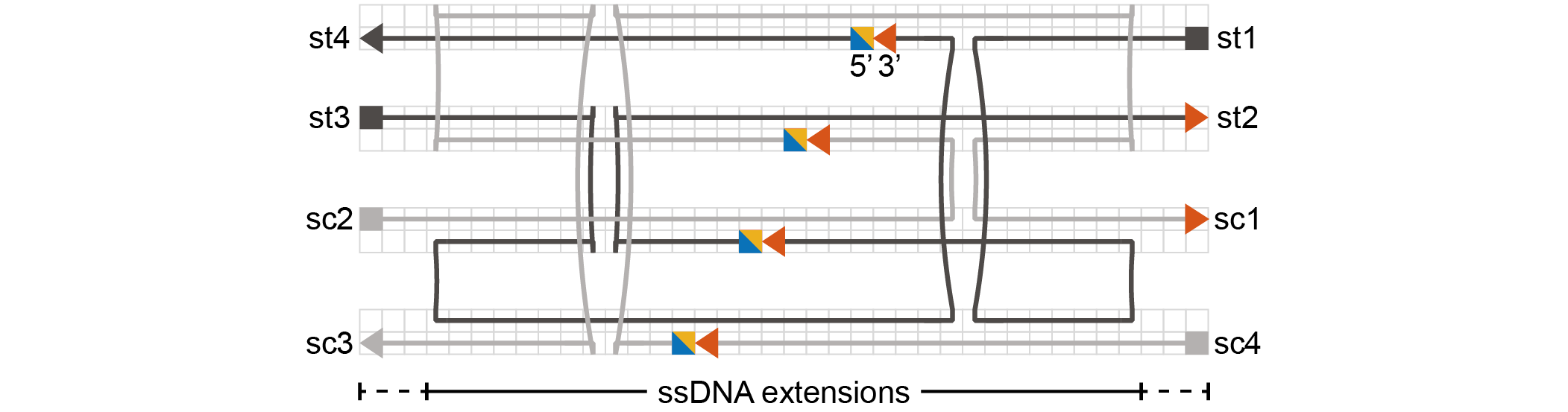


**Supplementary Figure S12.** Design and oligonucleotide pathways of DNA nanostructure. Grey background grid denotes one nucleotide per square. Single-stranded DNA extensions composed of three thymine nucleotides (TTT) prevent stacking interactions on each side of the design. For sequences refer to Supplementary Table S3.

**Supplementary Table S3.** DNA sequences employed for DNA nanostructures.

| **DNA strand** | **Sequence (5’ to 3’)** |
| --- | --- |
| sc1 | CCTTTCCACGAACACAGGGTTGTCCGATCCTATATTACGACTCCTTT |
| sc2 | TTTGGGAAGGGGTTCGCAAGTCGCACCCTAAACG |
| sc3 | TCTTATCCTGCATCGAAAGCTCAATCATGCATCTTT |
| sc4 | TTTATGTTGAAGGCTCAGGATGC |
| st1 | TTTATCGGACATTCAACATGGAGTCGTGGTGCGACT |
| st2 | TGCGAACAGGATAAGACGTTTAGAATATAGGTTT |
| st3 | TTTTTCGATGCCCCTTCCCGATGCATGAAGGGCATCCTGAGCCACCC |
| st4 | TGTGTTCGTGGAATTGAGCTTTT |


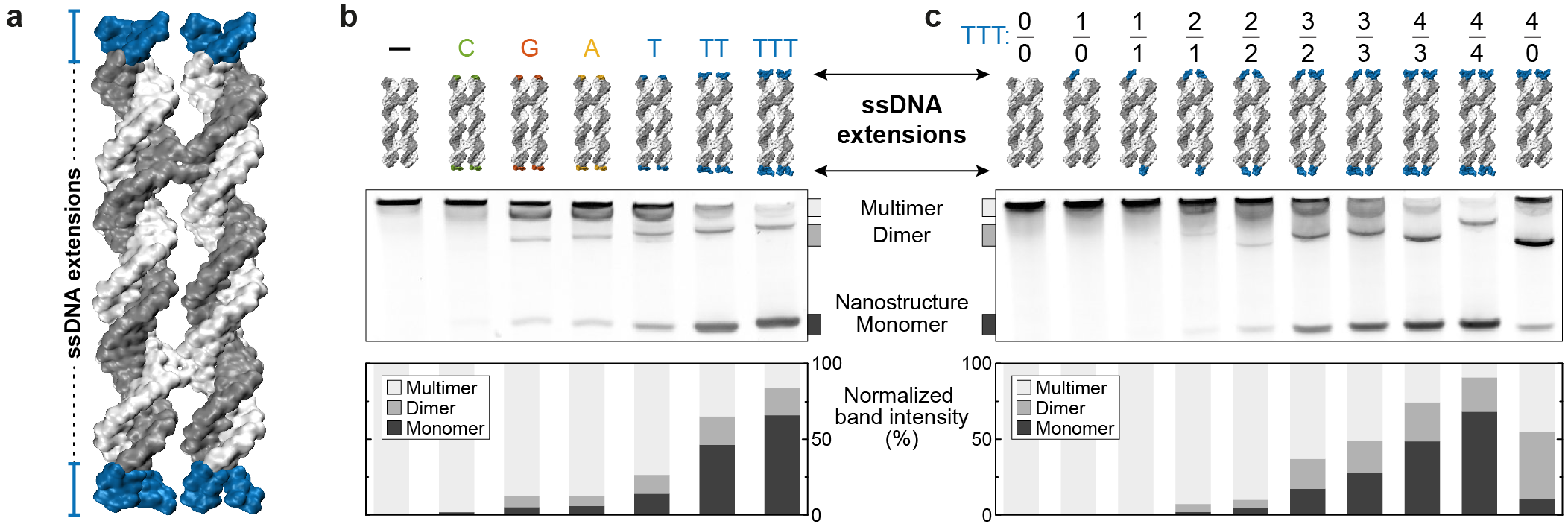


**Supplementary Figure S13.** Stacking prevention of DNA nanostructures with ssDNA extensions. **a** Side view of a 3D representation of the DNA nanostructure. Blue colored nucleotides highlight ssDNA extensions. **b** PAGE of DNA nanostructures assembled with different ssDNA extensions, ranging from one single nucleotide on all eight ends (C, G, A or T) to multiple thymidines (TT and TTT). Thymine nucleotides reduce stacking interactions best. **c** PAGE showing aggregation reduction by gradually increasing the number of TTT extensions on each side of the DNA nanostructure. Bottom histograms in (**b**) and (**c**) plot the intensities of the monomer, dimer, and multimer bands normalized to their sum per lane.


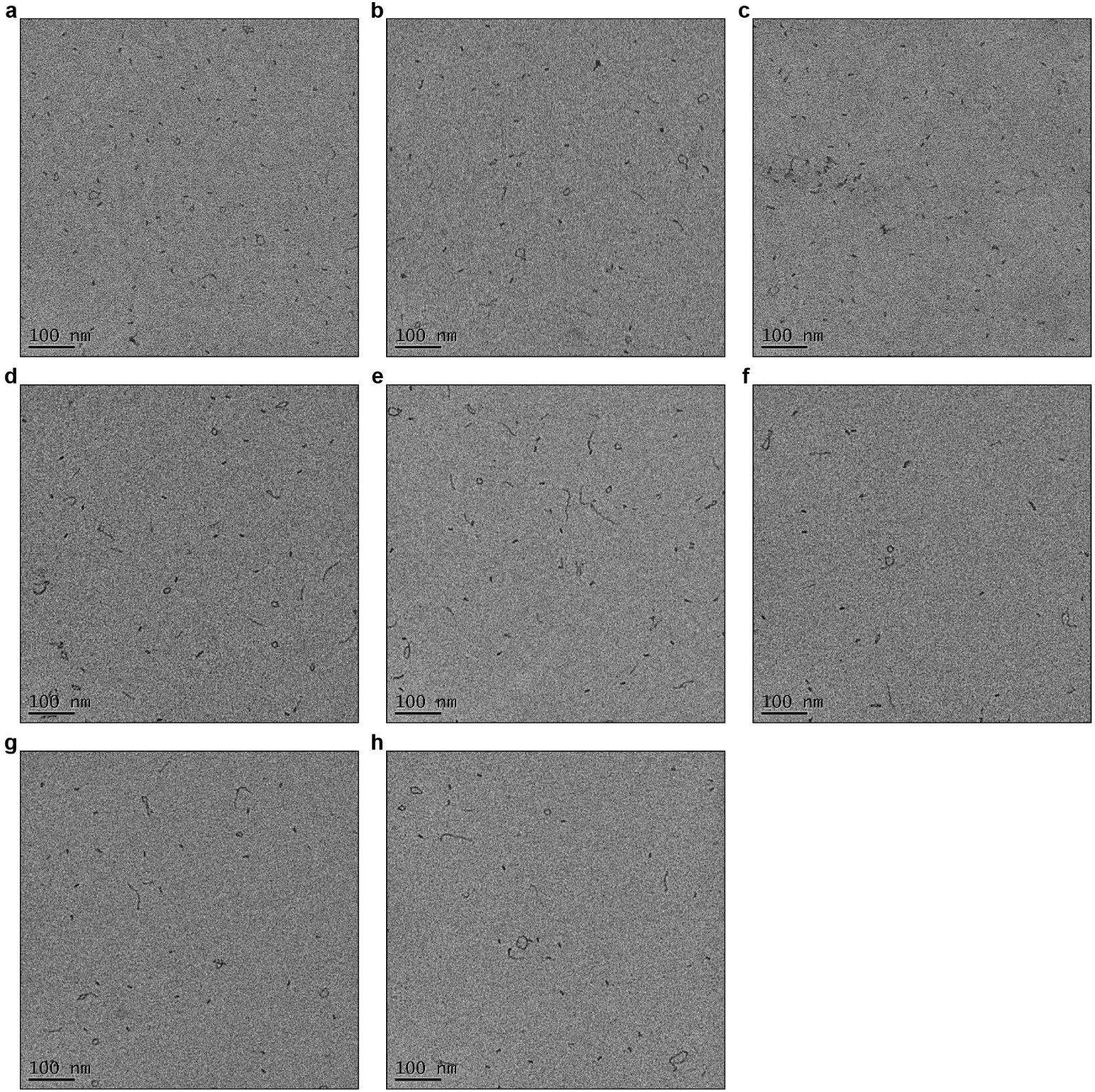


**Supplementary Figure S14.** Transmission electron microscopy images used for the size determination of DNA nanostructures assembled with all eight TTT ssDNA extensions.

**Supplementary Table S4.** DNA sequences of cholesterol-modified oligonucleotides used for DNA nanostructure assembly. Adenine spacer nucleotides (A) preceding the cholesterol modification are highlighted in green.

| **DNA strand** | **Sequence (5’ to 3’)** | **Supplier** |
| --- | --- | --- |
| sc4C_TEG | TTTATGTTGAAGGCTCAGGATGCA-CholTEG | IDT |
| sc2C_TEG | TTTGGGAAGGGGTTCGCAAGTCGCACCCTAAACGA-CholTEG | IDT |
| st3C_TEG | TTTTTCGATGCCCCTTCCCGATGCATGAAGGGCATCCTGAGCCACCCA-CholTEG | IDT |
| st1C_TEG | TTTATCGGACATTCAACATGGAGTCGTGGTGCGACTA-CholTEG | IDT |
| sc1C_TEG / C6 | CholTEG/C6-ACCTTTCCACGAACACAGGGTTGTCCGATCCTATATTACGACTCCTTT | Biomers |
| sc3C_TEG / C6 | CholTEG/C6-ATCTTATCCTGCATCGAAAGCTCAATCATGCATCTTT | Biomers |
| st4C_TEG / C6 | CholTEG/C6-ATGTGTTCGTGGAATTGAGCTTTT | Biomers |
| st2C_TEG / C6 | CholTEG/C6-ATGCGAACAGGATAAGACGTTTAGAATATAGGTTT | Biomers |
| sc1C_TEG_3’ | CCTTTCCACGAACACAGGGTTGTCCGATCCTATATTACGACTCCTTT-CholTEG | IDT |
| st2C_TEG_3’ | TGCGAACAGGATAAGACGTTTAGAATATAGGTTT-CholTEG | IDT |

**Supplementary Table S5.** DNA sequences employed for DNA nanostructures assembled with different number of cholesterol modifications and different linker types as shown in PAGE results in main text Figure 4E. All unmodified strands were composed of sequences shown in Supplementary Table S3.

| **# of cholesterol modifications** | **Linker type** | **DNA strands with cholesterol modifications** |
| --- | --- | --- |
| 0 | --- | --- |
| 1 | 3‘ TEG | sc4C_TEG |
| 2 | 3‘ TEG | sc4C_TEG, sc2C_TEG |
| 3 | 3‘ TEG | sc4C_TEG, sc2C_TEG, st3C_TEG |
| 4 | 3‘ TEG | sc4C_TEG, sc2C_TEG, st3C_TEG, st1C_TEG |
| 1 | 5‘ TEG | sc1C_TEG |
| 2 | 5‘ TEG | sc1C_TEG, sc3C_TEG |
| 3 | 5‘ TEG | sc1C_TEG, sc3C_TEG, st4C_TEG |
| 4 | 5‘ TEG | sc1C_TEG, sc3C_TEG, st4C_TEG, st2C_TEG |
| 1 | 5‘ C6 | sc1C_C6 |
| 2 | 5‘ C6 | sc1C_C6, sc3C_C6 |
| 3 | 5‘ C6 | sc1C_C6, sc3C_C6, st4C_C6 |
| 4 | 5‘ C6 | sc1C_C6, sc3C_C6, st4C_C6, st2C_C6 |

**Supplementary Table S6.** DNA sequences employed for DNA nanostructures assembled with different number and location of cholesterol modifications as shown in PAGE results in main text Figure 4F. All unmodified strands were composed of sequences shown in Supplementary Table S3.

| **Design** | **DNA strand with 3’ cholesterol** | **DNA strand with 5’ cholesterol** |
| --- | --- | --- |
| 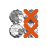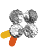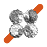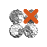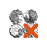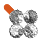1C_1 | sc4C_TEG | --- |
| 1C_2 | sc1C_TEG_3’ | --- |
| 1C_3 | st2C_TEG_3’ | --- |
| 2C_1 | sc4C_TEG, sc2C_TEG | --- |
| 2C_2 | sc1C_TEG_3’, st2C_TEG_3’ | --- |
| 2C_3 | sc4C_TEG | sc1C_TEG |


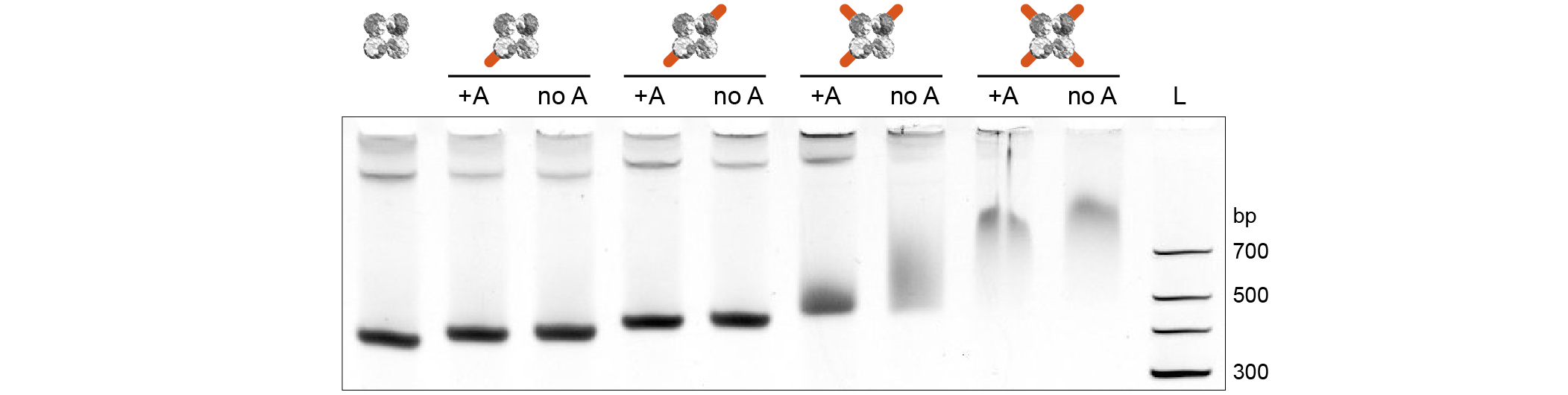


**Supplementary Figure S15.** PAGE image of DNA nanostructures assembled with 3’ TEG cholesterol tags at the indicated positions either with (+A) or without (no A) an additional adenine spacer nucleotide preceding the cholesterol modification.


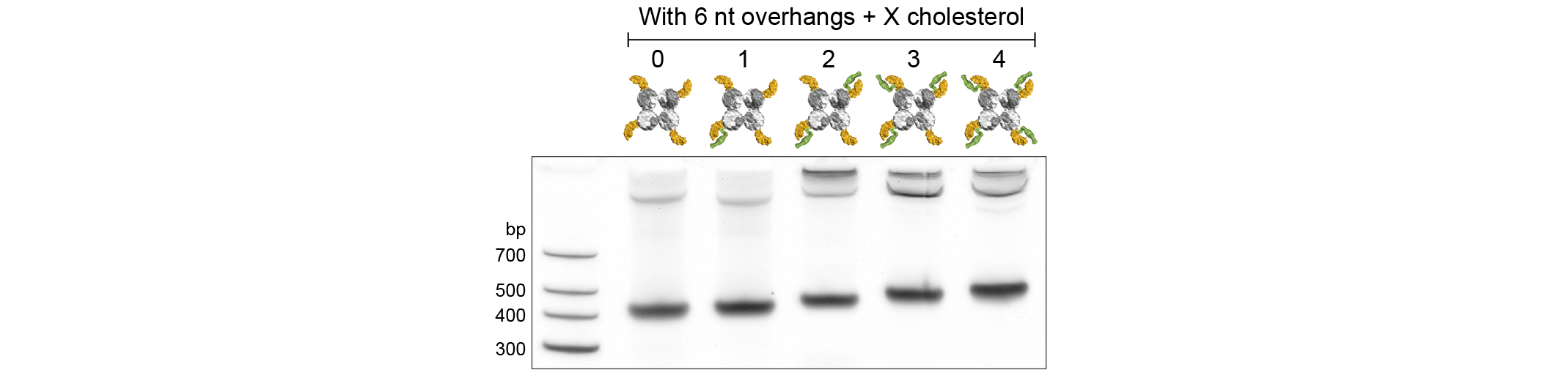


**Supplementary Figure S16.** PAGE analysis of DNA nanostructures assembled with 6 nt ssDNA overhangs and zero to four 3’ TEG cholesterol tags demonstrating consistent band shifts for each additional cholesterol group.

**Supplementary Table S7.** DNA sequences employed for Cy3-labeled DNA nanostructures assembled with four cholesterol modifications shielded by 6 nt overhangs (orange).

| **DNA strand** | **Sequence (5’ to 3’)** |
| --- | --- |
| sc1_6T | TTTTTTCCTTTCCACGAACACAGGGTTGTCCGATCCTATATTACGACTCCTTT |
| sc2_TEG | TTTGGGAAGGGGTTCGCAAGTCGCACCCTAAACGA-CholTEG |
| sc3_6T | TTTTTTTCTTATCCTGCATCGAAAGCTCAATCATGCATCTTT |
| sc4_TEG | TTTATGTTGAAGGCTCAGGATGCA-CholTEG |
| st1_TEG | TTTATCGGACATTCAACATGGAGTCGTGGTGCGACTA-CholTEG |
| st2_6T_Cy3 | TTTTTTTGCGAACAGGATAAGACGTTTAGAATATAGGTTT-Cy3 |
| st3_TEG | TTTTTCGATGCCCCTTCCCGATGCATGAAGGGCATCCTGAGCCACCCA-CholTEG |
| st4_6T_Cy3 | TTTTTTTGTGTTCGTGGAATTGAGCTTTT-Cy3 |


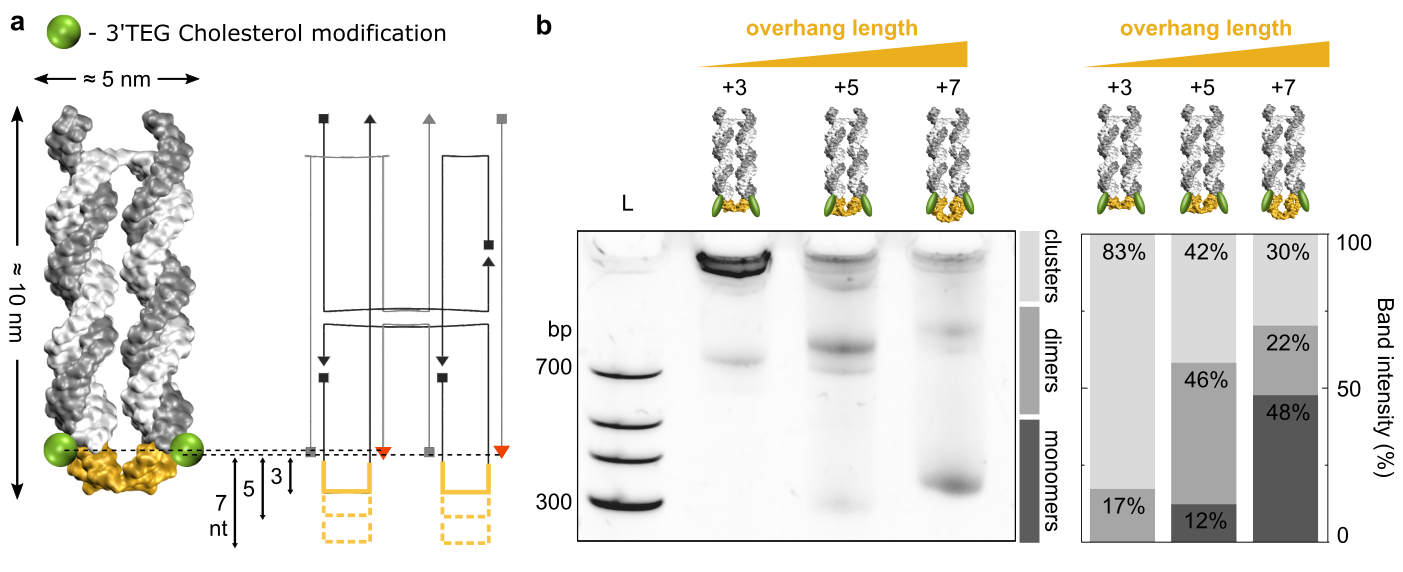


**Supplementary Figure S17.** Additional DNA nanostructure design demonstrating reduced aggregation with increased ssDNA overhang length. **a** Side view of a 3D model (left) and 2D design scheme (right) of another 4-helix DNA nanostructure highlighting cholesterol modification positions (green) and adjacent ssDNA loops (orange) of increasing overhang length to prevent aggregation. Overhangs are composed of poly-T. **b** PAGE of the DNA nanostructure with different overhang lengths (left). The histogram of the band intensities normalized to each lane (right) illustrates that the amount of monomers increases with increasing overhang length while clustering is at the same time reduced.

**Descriptions of Supplementary Movies**

**Supplementary Movie M1.** All-atom MD simulation of cholesterol-modified ssDNA. The movie illustrates the 0.5 µs MD trajectory of a 30 thymidine (30T) ssDNA strand. Two independent simulation runs starting from the same initial configuration are shown (Run 1 and Run 2). The ssDNA wraps around the cholesterol group throughout both simulation runs. Water and ions are not shown for clarity.

**Supplementary Movie M2.** All-atom MD simulation of a cholesterol-modified DNA duplex without overhang. The movie illustrates the 0.5 µs MD trajectory of a DNA duplex with one cholesterol tag but without an adjacent ssDNA overhang. Two independent simulation runs starting from the same initial configuration are shown (Run 1 and Run 2). Water and ions are not shown for clarity.

**Supplementary Movie M3.** All-atom MD simulation of a cholesterol-modified DNA duplex with a 6 nt overhang. The movie illustrates the 0.5 µs MD trajectory of a DNA duplex with one cholesterol tag and an adjacent 6 nt overhang. Two independent simulation runs starting from the same initial configuration are shown (Run 1 and Run 2). Water and ions are not shown for clarity.
